# Tailored Ion Release of Polycaprolactone and Calcium Silicate Composite Fibers Attenuates Neutrophil Extracellular Trap Formation for Dentin–Pulp Complex Regeneration

**DOI:** 10.34133/bmr.0388

**Published:** 2026-07-14

**Authors:** Jeong-Hyun Ryu, En Shi Jiang, Eunhye Lee, Jieun Bae, Wonjoon Moon, Shin Hye Chung, Kyung Mi Woo

**Affiliations:** ^1^ Department of Molecular Genetics, School of Dentistry, Seoul National University, Seoul, Republic of Korea.; ^2^ Department of Dental Biomaterials Science, Dental Research Institute, School of Dentistry, Seoul National University, Seoul, Republic of Korea.; ^3^ Department of Pharmacology & Dental Therapeutics, Dental Research Institute, School of Dentistry, Seoul National University, Seoul, Republic of Korea.

## Abstract

While calcium silicate (CS) remains a gold standard material for vital pulp therapy, its clinical efficacy is frequently compromised by an abrupt ion release during the initial setting phase that induces extreme alkalinity and acute cytotoxicity. We engineered electrospun poly-ε-caprolactone (PCL)/CS composite fibers to reconfigure CS hydration kinetics, achieving sustained and controlled ion release. In vitro evaluation using phase-specific eluates and defined pH/Ca^2+^ conditions demonstrated that CS eluates from the initial setting phase induced significant cytotoxicity and excessive NETosis in primary neutrophils, whereas PCL/CS eluates maintained both at control levels. Mechanistically, neutrophil extracellular traps (NETs) isolated from CS-stimulated neutrophils directly drove M1 macrophage polarization, while PCL/CS eluates promoted an anti-inflammatory and proregenerative macrophage phenotype. PCL/CS eluates, conditioned media from PCL/CS-treated macrophages, and PCL/CS nanofibrous substrates all promoted odontoblastic differentiation. In vivo validation using a rat molar pulp exposure model revealed that during the early host response (at days 2 and 7), CS provoked marked neutrophil infiltration and excessive NETosis, whereas PCL/CS attenuated the acute inflammatory response and promoted timely inflammatory resolution and M2 macrophage polarization. At 8 weeks, the PCL/CS group exhibited improved dentin–pulp regeneration characterized by organized tubular dentin and polarized odontoblasts, rather than amorphous osteodentin typically observed with CS. PCL/CS also mitigated furcal bone resorption. These findings indicate that immune modulation through tailored ion release via the NETosis–macrophage axis represents an effective strategy for supporting dentin–pulp complex regeneration.

## Introduction

Bioinorganic materials—such as calcium phosphates, bioactive glasses, and calcium silicates (CSs)—are increasingly incorporated into polymer-based scaffolds to enhance their regenerative functionality in tissue engineering [1,2]. However, the uncontrolled burst release of inorganic ions can generate supraphysiological ion concentrations and, in the case of CS, extreme local alkalinity, provoking acute cytotoxicity and aberrant innate immune responses [3,4]. Tight regulation of ion release kinetics is therefore essential for translating bioinorganic strategies into clinical practice. Among bioinorganic materials, CSs represent the clinical gold standard for vital pulp therapy owing to their capacity to induce reparative dentin formation [5–8]; however, this benefit is offset by the physicochemical insult inherent to their setting reaction. Dental pulp exposure, frequently encountered in dental caries and trauma, necessitates direct pulp capping to protect the pulp and preserve the integrity of the dentin–pulp complex. Hydration of CS generates CS hydrate and calcium hydroxide (Ca(OH)_2_), resulting in the rapid release of calcium and hydroxyl ions that elevates the local pH to approximately 12.5 and produces supraphysiological calcium during the early setting phase [9,10]. This acute ion surge is directly cytotoxic, markedly reducing the viability of pulp-adjacent cells in vitro [3,4] and correlating with persistent postoperative pain in clinical reports [11]. Moreover, CS-induced cell death depletes the local progenitor pool essential for regeneration and releases intracellular contents that act as damage-associated molecular patterns (DAMPs), further amplifying the inflammatory response [3,4,12]. These limitations highlight the need for biomaterial strategies capable of modulating the early inflammatory microenvironment while preserving the regenerative benefits of CS.

Beyond direct cytotoxicity, the physicochemical microenvironment generated by CS hydration has important implications for innate immune responses, particularly NETosis. Neutrophils are the first immune cells recruited to injured tissue and play a critical role in the initial inflammatory phase of wound healing. Under pathological stimuli, neutrophils can undergo NETosis, a distinct form of cell death characterized by the release of neutrophil extracellular traps (NETs)—weblike structures composed of decondensed chromatin decorated with granule proteins such as neutrophil elastase (NE) and myeloperoxidase [13]. Importantly, elevated extracellular calcium concentrations have been shown to directly trigger NETosis through calcium-influx-dependent signaling pathways [14–16]. Additionally, alkaline conditions have been shown to further augment NETosis by enhancing peptidyl arginine deiminase 4 (PAD4)-mediated histone citrullination and promoting chromatin decondensation [17,18]. These findings suggest that the physicochemical microenvironment generated by CS hydration—characterized by supraphysiological calcium concentrations and extreme alkalinity—may serve as a potent trigger for excessive NETosis at the pulp–capping material interface. Although NETosis initially serves a protective antimicrobial function, excessive or sustained NET formation disrupts the orderly progression of wound healing. NETs and their associated granule components, including NE and histones, act as secondary DAMPs that perpetuate inflammation by activating macrophages toward a pro-inflammatory M1 phenotype and inhibiting the phenotypic transition to the anti-inflammatory, proregenerative M2 state, thereby delaying the resolution of inflammation and impairing subsequent tissue repair [19,20]. The importance of this M1-to-M2 transition in the context of dentin–pulp regeneration has been increasingly recognized; M2 macrophages secrete anti-inflammatory cytokines that promote odontoblast differentiation and reparative dentin formation [21,22], while M1-dominant environments sustain inflammation and impair pulp tissue regeneration. Therefore, modulating the early NETosis–macrophage axis may represent an effective strategy for creating an immunomodulatory microenvironment favorable for dentin–pulp regeneration.

Nanofibrous scaffolds offer a promising platform for modulating both the physicochemical and topographical microenvironment at the material–tissue interface. Their high surface-area-to-volume ratio and nanoscale surface architecture enhance protein adsorption—particularly of fibronectin and vitronectin—which in turn promotes integrin-mediated focal adhesion assembly and downstream signaling cascades that influence cell adhesion, spreading, and differentiation [23–25]. Indeed, nanotopographic cues have been shown to direct stem cell fate through mechanosensitive pathways involving focal adhesion kinase and cytoskeletal remodeling [26]. In the context of dental tissue engineering, nanofibrous topography has been shown to facilitate mesenchymal stem cell recruitment through M2 macrophage-mediated paracrine signaling [27] and to direct odontoblastic differentiation of dental pulp stem cells through Wnt-signaling-mediated pathways [28–30]. Poly-ε-caprolactone (PCL), a Food and Drug Administration-approved aliphatic polyester with established biocompatibility and slow biodegradation [31], provides a suitable polymer matrix for incorporating CS particles. Notably, the hydrophobic nature of the PCL matrix can physically restrict water access to embedded CS particles, thereby modulating their hydration kinetics and attenuating the initial burst release of ions. Such polymer-mediated control of inorganic ion release has been widely demonstrated in composite scaffold systems, where the polymer matrix serves as a diffusion barrier that converts an otherwise abrupt burst into a sustained, therapeutically effective release profile [32,33]. Furthermore, the therapeutic versatility of functionalized PCL nanofibers has been demonstrated in the in situ regeneration of infected developing dental roots, where they promoted M2 macrophage polarization and tissue development [34].

Although electrospun PCL/CS composites, PCL/bioactive-glass fibers, and other ion-release-controlled scaffolds have been extensively investigated for dental tissue engineering [35,36], their development has focused largely on physicochemical optimization and direct regenerative performance rather than on harnessing controlled ion release to modulate the early NETosis–macrophage response. Based on these considerations, we hypothesized that incorporating CS into an electrospun PCL nanofibrous matrix would reconfigure CS hydration kinetics, enabling a sustained and controlled ion release that mitigates the acute cytotoxic and pro-inflammatory insult associated with conventional CS. We further hypothesized that this tailored ion release would attenuate excessive NETosis, thereby facilitating a favorable shift toward M2 macrophage polarization and ultimately promoting dentin–pulp complex regeneration. To test these hypotheses, we fabricated PCL/CS composite fibers by electrospinning and characterized their physicochemical properties, including crystallization behavior, ion release kinetics, and mechanical integrity. We then evaluated their biological effects in vitro, examining cytotoxicity and NETosis induced by CS and PCL/CS eluates as well as by varying pH and calcium ion concentrations, macrophage polarization in response to material eluates and isolated NETs, and odontoblast differentiation mediated by the eluates and macrophage-conditioned media (CM). Finally, in vivo validation was performed using a rat molar pulp exposure model to assess early immune modulation and tissue regeneration outcomes.

## Materials and Methods

### Experimental design

The primary objective of this study was to develop an immunomodulatory PCL/CS composite scaffold and evaluate its efficacy in supporting dentin–pulp complex regeneration by attenuating early inflammatory responses. The study design consisted of 4 integrated phases: (1) fabrication and physicochemical characterization of electrospun PCL/CS composite fibers, including morphological analysis (scanning electron microscopy coupled with energy-dispersive x-ray spectroscopy [SEM–EDS]), crystallographic assessment (x-ray diffraction [XRD]), mechanical evaluation (tensile strength), and surface wettability (contact angle); (2) characterization of ion release properties, including sustained release profiles of Ca^2+^ and Si ions measured by inductively coupled plasma optical emission spectroscopy (ICP-OES) and pH monitoring over time; (3) in vitro evaluation of biological responses, comprising 3 sequential sub-phases: (3a) cytotoxicity and NETosis assessment in primary neutrophils and MDPC-23 pre-odontoblasts under CS and PCL/CS eluates as well as varied pH and calcium ion concentrations, (3b) assessment of macrophage polarization in response to CS and PCL/CS eluates and isolated NETs, evaluated by M1/M2 marker expression via quantitative real-time polymerase chain reaction (RT-qPCR), and (3c) evaluation of odontoblastic differentiation through eluate exposure, macrophage-CM, and substrate contact; and (4) in vivo validation using a rat molar pulp exposure model, conducted at 2 sequential time points: (4a) early immune responses at 2 and 7 d post-operation, assessed by histological and immunohistochemical analyses of neutrophil infiltration, NETosis, and macrophage polarization, and (4b) tissue regeneration at 4 and 8 weeks post-operation, evaluated by micro-computed tomography (micro-CT) and histology for dentin bridge formation and furcal bone integrity. Statistical comparisons between CS and PCL/CS groups were performed throughout all phases. The overall conceptual workflow, including materials, treatments, and measurements, is illustrated in Fig. S1.

### Fabrication of electrospun PCL/CS composite fibers

PCL fibrous membranes were fabricated using the electrospinning method (Fig. S2). Briefly, 1.2 g of PCL was dissolved in 4 ml of dichloromethane for 1 h, after which 6 ml of dimethylformamide was added to yield a final polymer concentration of 12% (w/v). For PCL/CS composite fibrous membranes, 1.2 g of CS particles (ProRoot MTA; Dentsply Sirona, Charlotte, NC, USA) was first dispersed in 6 ml of dimethylformamide by ultrasonication for 10 min and then combined with PCL predissolved in 4 ml of dichloromethane. The mixed solution was loaded into a 10-ml syringe fitted with a 21-gauge needle and electrospun onto a rotating drum collector (drum diameter, 15 cm; rotation rate, 460 rpm). The electric potential and tip-to-collector distance were 15 kV and 15 cm, respectively. To prepare the hydraulically set form of PCL/CS fibrous membranes for subsequent cell culture and animal studies, the as-spun (unset) PCL/CS membranes were immersed in distilled water at room temperature to allow hydration and initial hydraulic setting of the embedded CS particles, after which the membranes were retrieved and dried to obtain preset PCL/CS membranes.

### Physicochemical characterization of PCL/CS composite fibers

The morphology and elemental composition of PCL/CS composite fibers were examined by SEM–EDS (Auriga, Carl Zeiss, Jena, Germany). Both unset PCL/CS and hydrated (preset) PCL/CS specimens were sputter-coated with platinum at 20 mA for 100 s prior to analysis. Fiber diameters were measured from SEM images using the ImageJ software (National Institutes of Health, Bethesda, MD, USA); at least 100 fibers per specimen were measured.

Tensile strength and elastic modulus were determined using a universal testing machine (model 5942; Instron, Norwood, MA, USA). Specimens of PCL, unset PCL/CS, and (hydrated) PCL/CS were cut to 10 × 60 mm^2^ (width × length) and tested at a crosshead speed of 5 mm/min. Six specimens were tested per group, and mean values with standard deviations (SDs) were reported.

The water contact angles of PCL, unset PCL/CS, and PCL/CS fibers were measured by the sessile drop method at room temperature using an optical contact angle goniometer (Femtofab, Seongnam, Korea). The left and right contact angles of each droplet were averaged.

XRD analysis was performed to evaluate the crystalline phases at each stage of the manufacturing process. CS and PCL/CS specimens were analyzed at 3 stages: as-received/unset, hydrated (immersed in distilled water for 24 h), and water-immersed (hydrated specimens further immersed in distilled water for an additional 3 d). XRD patterns were recorded on a D8 Advance with DaVinci (Bruker Optics, Ettlingen, Germany) using Cu Kα radiation (*λ* = 1.5418 Å; 40 kV, 40 mA) and a LynxEye XE detector. Diffraction patterns were collected over a 2*θ* range of 10° to 80°, with a measuring time of 0.5 s per step. Measurements were performed in triplicate for each condition, and representative patterns are presented.

### pH and ion release analysis

The pH and ion release profiles were characterized using eluates obtained from hydrated CS and PCL/CS. For pH measurement, CS (2 g of powder) and PCL/CS (15-cm^2^ membrane) were each immersed in 10 ml of phosphate-buffered saline (PBS; pH 7.4). Specimens were incubated at 37 °C, and the immersion medium was collected and replaced with fresh PBS at 1, 2, 3, 7, and 14 d. The pH of the collected eluates was measured using a pH meter (Orion Star A211; Thermo Fisher Scientific, Waltham, MA, USA).

For calcium and silicon ion release analysis, CS (2 g of powder) and PCL/CS (15-cm^2^ membrane) were each immersed in 10 ml of double-distilled water (DDW), and the immersion medium was collected and replaced at 1, 2, 3, 7, and 14 d. The collected eluates were filtered through a 0.22-μm syringe filter and analyzed for calcium and silicon ion concentrations by ICP-OES (Agilent 5800, Agilent Technologies, Palo Alto, CA, USA). The filtered eluates were also reserved for subsequent cell culture experiments. Because CS and PCL/CS differ in physical form, a direct mass-based comparison is not straightforward; therefore, the amount of each material immersed was scaled to maintain the proportion of each material applied per treatment in the rat pulp–capping model.

### Cell culture

The MDPC-23 cell line, derived from mouse fetal molar dental papillae [37], was cultured in Dulbecco’s modified Eagle medium (DMEM; HyClone, Logan, UT, USA) supplemented with 10% fetal bovine serum (FBS; Gibco BRL, Grand Island, NY, USA) and 1% penicillin–streptomycin. The odontoblastic differentiation medium was prepared by adding 50 μg/ml ascorbic acid and 10 mM β-glycerophosphate to the growth medium.

Mouse primary neutrophils were isolated from long bone marrows by density gradient centrifugation using Histopaque-1077 and Histopaque-1119 (Sigma-Aldrich, St. Louis, MO, USA) [38] and cultured in RPMI 1640 medium containing HEPES and l-glutamine (HyClone) supplemented with 10% FBS (Gibco BRL) and 1% penicillin–streptomycin.

Mouse primary bone-marrow-derived macrophages (BMDMs) were isolated from long bone marrows using the same density gradient procedure as described for neutrophils. Cells were seeded at a density of 5 × 10^5^ cells per well into 12-well plates and differentiated in the presence of macrophage colony-stimulating factor (M-CSF; 40 ng/ml; PeproTech, Rocky Hill, NJ, USA) for 7 d, with medium replacement every 2 d.

### Cytotoxicity assay

Primary neutrophils and MDPC-23 pre-odontoblasts were treated with CS or PCL/CS eluate-containing media (25% or 50% v/v) or exposed to defined pH conditions and culture medium (basal Ca^2+^: 1.8 mM) supplemented with additional CaCl_2_ (5, 10, or 20 mM). Powdered DMEM was reconstituted with the DDW-based eluate in place of water, so that the resulting medium contained the eluate-derived ions at the designated concentration (25% or 50% v/v). For primary neutrophils, 1 × 10^5^ cells per well were seeded into black-wall, clear-bottom 96-well plates and incubated with each treatment condition in the presence of SytoxGreen (Invitrogen, Carlsbad, CA, USA) at a final concentration of 1 μM. After 1 h of incubation, fluorescence intensity was measured at excitation/emission wavelengths of 485/535 nm using a microplate reader (SPARK 10M; Tecan, Männedorf, Switzerland). For MDPC-23 cells, 1.5 × 10^4^ cells per well were seeded into 96-well plates and allowed to attach overnight. The following day, cells were treated under the same conditions for 6 h, followed by incubation with SytoxGreen (1 μM) for 10 min. Fluorescence intensity was then measured at 485/535 nm.

### NETosis assay

Primary neutrophils were seeded into poly-d-lysine-coated μ-Plate 96-Well Square plates (ibidi, Gräfelfing, Germany) at a density of 1 × 10^5^ cells per well and allowed to stabilize for 1 h. Cells were then treated with CS or PCL/CS eluate-containing media (25% or 50% v/v) or exposed to defined pH conditions and culture medium supplemented with additional CaCl_2_ (5, 10, or 20 mM) as described above. After 4 h of incubation, cells were fixed with 4% paraformaldehyde (PFA) for 10 min at room temperature and permeabilized with 0.1% Triton X-100 in PBS for 5 min. Cells were blocked with 5% normal donkey serum in tris-buffered saline (TBS) containing 0.1% Tween 20 (TBS-T) for 1 h at room temperature and then incubated with a primary antibody against citrullinated histone H3 (CitH3; Abcam, Cambridge, UK) at a dilution of 1:200 for 1 h at room temperature. After washing, cells were incubated with an Alexa Fluor 488-conjugated secondary antibody (Invitrogen) at a dilution of 1:500 for 1 h at room temperature. Nuclei were counterstained with 4′,6-diamidino-2-phenylindole (DAPI; 5 μM) for 5 min. Fluorescence images were acquired using a confocal laser scanning microscope (LSM 800; Carl Zeiss, Oberkochen, Germany). The CitH3-positive area was quantified using the ImageJ software and normalized to the DAPI-stained area. Primary cells were isolated from 3 independent animals, and experiments were independently repeated for each animal.

### Flow cytometric analysis of NETosis

To corroborate the CitH3 immunostaining results, SytoxGreen-based flow cytometric analysis was performed. Primary neutrophils were treated with CS or PCL/CS eluate-containing media (50% v/v) or calcium ionophore A23187 (positive control) for 4 h. Cells were stained with SytoxGreen (1 μM) and analyzed by flow cytometry. The fluorescence shift pattern was compared across treatment conditions. Data were acquired using a BD FACSAria Fusion Cell Sorter (BD Biosciences, San Jose, CA, USA) and analyzed with the BD FACSDiva software.

### Isolation of NETs

Primary neutrophils were seeded at a density of 1 × 10^6^ cells per well into 12-well plates and stimulated with eluate-containing media (50% v/v) supplemented with 1% FBS for 4 h; low serum was used to minimize serum-mediated degradation of NETs [20]. Following stimulation, both supernatants and adherent NETs were collected by gentle scraping. The collected suspension was centrifuged at 300 × g for 5 min at 4 °C to remove intact cells and debris. The resulting supernatant was further centrifuged at 18,000 × g for 10 min at 4 °C to pellet the NETs. The NET pellet was resuspended in serum-free DMEM for subsequent experiments.

### Macrophage polarization analysis

On day 6 of the 7-d differentiation period, BMDMs were treated with CS or PCL/CS eluates (25% v/v) or NETs isolated from eluate-stimulated neutrophils. For NET treatment, NETs derived from 1 × 10^6^ neutrophils were applied to 5 × 10^5^ macrophages. Cells were cultured in DMEM supplemented with 10% FBS and M-CSF (40 ng/ml; PeproTech) for 24 h, so that harvesting coincided with the completion of differentiation at day 7. After incubation, cells were harvested for RT-qPCR analysis of M1 and M2 macrophage polarization markers, and the corresponding supernatants were collected for the preparation of macrophage-CM. Supernatants were centrifuged at 300 × g for 5 min to remove cells and debris and then filtered through a 0.22-μm syringe filter.

### Odontoblastic differentiation of MDPC-23 cells

MDPC-23 cells were seeded into 12-well plates at a density of 8 × 10^4^ cells per well. The following day, the medium was replaced with a differentiation medium containing CS or PCL/CS eluates (25% v/v) or macrophage-CM (1:5 dilution). The medium was refreshed every 2 d, and cells were cultured for up to 7 d.

For material-contact experiments, MDPC-23 cells were seeded on tissue culture dishes (TCD; control), PCL, CS, and PCL/CS substrates into 6-well plates at a density of 1 × 10^5^ cells per well and cultured in the differentiation medium for 7 d.

### Alkaline phosphatase staining and activity assay

For alkaline phosphatase (ALP) staining, MDPC-23 cells were seeded into 96-well plates at a density of 5 × 10^3^ cells per well. The following day, the medium was replaced with odontoblastic differentiation medium supplemented with CS or PCL/CS eluates (25% v/v) or macrophage-CM (1:5 dilution). The medium was refreshed every 2 d. After 7 d of culture, cells were fixed and stained with a 5-bromo-4-chloro-3-indolyl-phosphate/nitro blue tetrazolium (BCIP/NBT) substrate (Sigma-Aldrich) according to the manufacturer’s instructions. Stained images were acquired using a light microscope, and the ALP-stained area was quantified using the ImageJ software.

For ALP activity measurement under material-contact conditions, MDPC-23 cells were cultured on TCD (control), PCL, CS, and PCL/CS substrates as described above. After 7 d of culture, cells were lysed and analyzed using a SensoLyte pNPP ALP assay kit (AnaSpec, San Jose, CA, USA) according to the manufacturer’s instructions. ALP activity was normalized to the total protein content, which was determined using a Pierce BCA protein assay kit (Thermo Fisher Scientific).

### RNA extraction and quantitative real-time PCR

For in vitro experiments, total RNA was extracted using RNAiso Plus (Takara Bio, Otsu, Japan), and complementary DNA was synthesized using a PrimeScript RT reagent kit (Takara Bio). For in vivo experiments, coronal pulp tissues were carefully dissected from rat mandibular first molars at 7 d post-operation, and total RNA was extracted using RNAiso Plus. RT-qPCR was performed on a StepOnePlus Real-Time PCR system (Applied Biosystems, Foster City, CA, USA). Gene expression levels were normalized to glyceraldehyde-3-phosphate dehydrogenase as an internal control, except for macrophage assays, in which ribosomal protein lateral stalk subunit P0 (Rplp0) was used. The primer sequences are listed in Table S1.

### Animals and surgical procedures

All animal experiments were approved by the Institutional Animal Care and Use Committee of Seoul National University (SNU-170929-2). Specific pathogen-free male Sprague–Dawley rats (10 weeks old, 300 to 350 g; Orient Bio, Seongnam, Korea) were randomly divided into 2 groups (CS and PCL/CS). Rats were allowed to acclimate for 1 week prior to surgery. Anesthesia was induced by intraperitoneal injection of a mixture of tiletamine–zolazepam (Zoletil; 0.5 ml/kg; Virbac, Carros, France) and xylazine (Rompun; 0.7 mg/kg; Bayer, Leverkusen, Germany). Under 3.8× surgical loupes (SurgeLoup; Crystal Optic, Incheon, Korea), a pinpoint pulp exposure was made on the occlusal surface of the mandibular first molars using a round bur (diameter, 0.6 mm). The exposed pulp was irrigated with sterile saline, and hemostasis was achieved with a saline-soaked cotton pellet. The exposed site was then capped with CS or PCL/CS and sealed with a glass ionomer cement (Fuji II; GC America, Alsip, IL, USA) (Fig. S3). For the CS group, a moist cotton pellet was placed over the CS prior to glass ionomer sealing; the cotton pellet was removed the following day to confirm setting, and the cavity was resealed with glass ionomer. The opposing maxillary first molar was extracted to prevent occlusal trauma to the treated tooth. Animals were sacrificed at 2 d, 7 d, 4 weeks, and 8 weeks postsurgery by CO_2_ inhalation (*n* = 3 to 4 per group per time point for histological and micro-CT analyses; *n* = 4 per group at day 7 for RT-qPCR of coronal pulp tissues). Animals that died during the observation period were excluded from the analysis. Sample sizes were determined based on prior studies using similar pulp exposure models.

### Micro-CT analysis

Mandibular bone blocks containing the first molar were dissected and fixed in 4% PFA. Specimens were scanned using a high-resolution micro-CT system (SkyScan 1172; Bruker, Kontich, Belgium) at 100 kV/100 μA with a voxel size of 10 μm, a 0.5-mm aluminum filter, and 360° of rotation. Raw projection data were reconstructed into 3-dimensional images using the NRecon software (Bruker) with beam hardening correction set at 40% and lower/upper gray thresholds of 65 and 255, respectively. Reconstructed data were reformatted using CTVol (Bruker). Volumetric analyses were performed using the CTAn software (Bruker) to determine the volumes of calcified tissue within the pulp chamber and the bone volume fraction (BV/TV) at the furcation area. The furcal volumetric analysis was restricted to the coronal 1 mm of alveolar bone measured from the furcation roof, based on the rationale that pulpal inflammation initially spreads to the coronal furcal region through accessory canals. All micro-CT analyses were performed in a blinded manner.

### Histological analysis and neutrophil quantification

Specimens were fixed in 4% PFA for 24 h at 4 °C and decalcified in 10% ethylenediaminetetraacetic acid solution for 1 week, followed by dehydration and paraffin embedding. Serial sagittal (mesiodistal) sections of 4-μm thickness were obtained. Histological analysis was performed on specimens collected at 2 d, 7 d, and 8 weeks postsurgery. Sections were stained with hematoxylin and eosin for the evaluation of tissue morphology. Slides were digitally scanned using a Panoramic MIDI scanner (3DHISTECH, Budapest, Hungary), and images were analyzed using the CaseViewer software (3DHISTECH). For neutrophil quantification, Giemsa staining (1:20 dilution, 30 min; Sigma-Aldrich) was performed on adjacent sections. Three square regions of interest (ROIs) of identical size were positioned within the pulp tissue adjacent to the exposure site at 100× magnification, with a consistent ROI size across all groups. Neutrophils were identified by their characteristic multilobed nuclei and quantified as the percentage of neutrophils relative to the total number of cells within each ROI. The average value from 3 ROIs per specimen was used for statistical analysis. All histological analyses were performed in a blinded manner.

### Immunofluorescence staining and quantification

For immunofluorescence staining, tissue sections were deparaffinized and subjected to heat-induced antigen retrieval in citrate buffer (pH 6.0) for 15 min. Slides were washed twice with TBS-T for 5 min and blocked with 1% bovine serum albumin in TBS for 2 h at room temperature. Sections were incubated overnight at 4 °C with primary antibodies against NE (1:200; Abcam), CitH3 (1:200; Abcam), CCR7 (1:200; Santa Cruz Biotechnology, Dallas, TX, USA), or CD163 (1:200; Santa Cruz Biotechnology). After washing, sections were incubated with Alexa Fluor 488- or 647-conjugated secondary antibodies (1:500; Invitrogen). Nuclei were counterstained with DAPI. For macrophage identification, sections were additionally stained with an anti-F4/80 (Adgre1) antibody (1:500; Santa Cruz Biotechnology). Fluorescence images were acquired using a confocal laser scanning microscope (LSM 800; Carl Zeiss, Oberkochen, Germany). For quantitative analysis of NETosis markers, 3 ROIs of identical dimensions were randomly positioned within the pulp horn area adjacent to the exposure site in each section. Image-based quantification was performed using the ImageJ software with a uniform threshold applied across all images. The positively stained area for NE or CitH3 was normalized to the SytoxGreen- or DAPI-stained area within the same ROI and expressed as the ratio of marker-positive area to the corresponding nuclear-stained area. For macrophage polarization analysis, CCR7–F4/80 and CD163–F4/80 double-positive cells were counted within each ROI, and the ratio of CD163–F4/80 double-positive cells to CCR7–F4/80 double-positive cells was calculated.

### Statistical analysis

Data are presented as mean ± SD. Normality was assessed using the Shapiro–Wilk test prior to parametric analyses. Statistical analyses were conducted using one-way analysis of variance with Tukey’s post hoc test for multigroup comparisons or the Mann–Whitney *U* test for animal experiments. Differences were considered statistically significant at *P* < 0.05.

## Results and Discussion

### Characterization of PCL/CS composite fibers

**Fig. 1.**
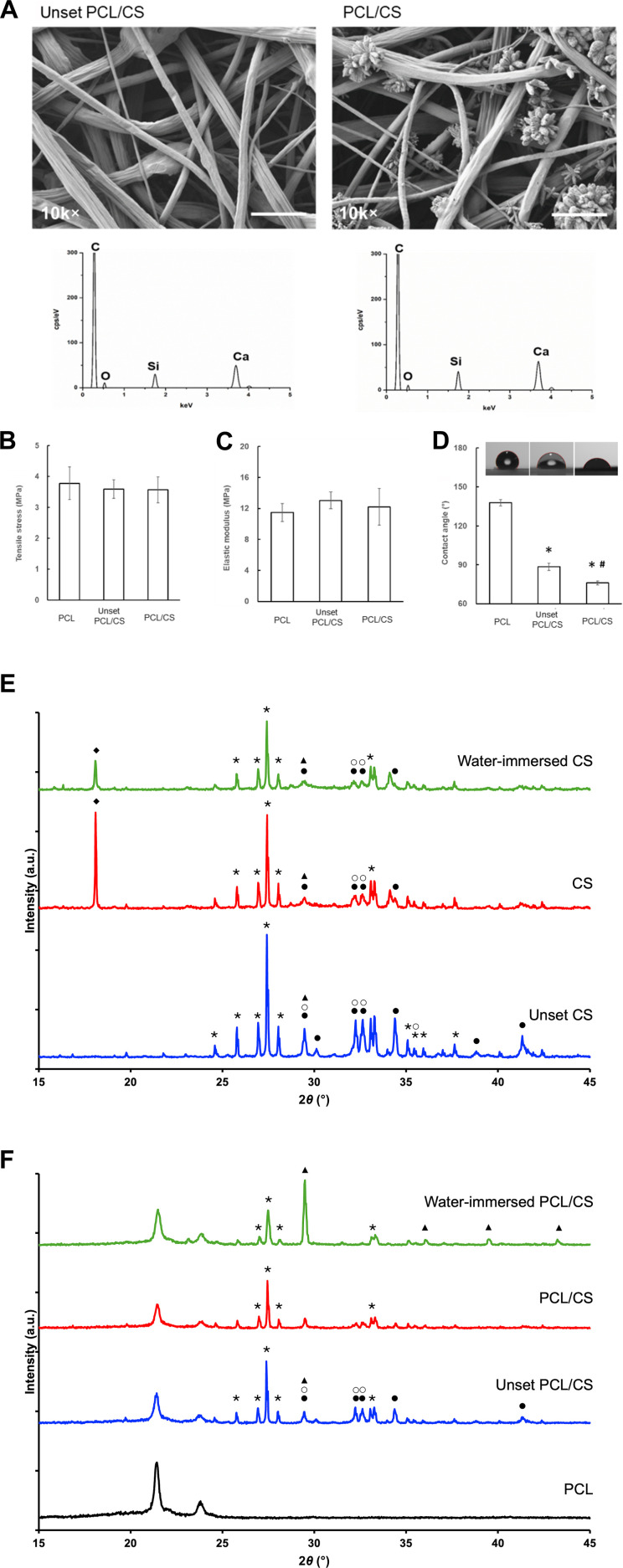
Physicochemical characterization of poly-ε-caprolactone (PCL)/calcium silicate (CS) composite fibers. (A) Scanning electron microscopy (SEM) views (top) and energy-dispersive x-ray spectroscopy (EDS) spectra (bottom) of unset PCL/CS and PCL/CS. Scale bar = 5 μm. (B) Tensile strengths of PCL, unset PCL/CS, and PCL/CS fibers. (C) Elastic moduli of PCL, unset PCL/CS, and PCL/CS fibers. (D) Contact angles of PCL, unset PCL/CS, and PCL/CS fibers. Data are presented as mean ± SD (*n* = 6). *P < 0.05 versus PCL; ^#^*P* < 0.05 versus unset PCL/CS. (E) XRD patterns of unset CS, CS, and immersed CS. (F) X-ray diffraction (XRD) patterns of PCL, unset PCL/CS, PCL/CS, and immersed PCL/CS. Diffraction patterns were collected within the 2*θ* range of 10° to 80°. XRD measurements were performed in triplicate for each condition, and representative patterns are shown.

The morphology of electrospun PCL/CS composite fibers was analyzed using SEM–EDS (Fig. 1A). The fibers exhibited a nano- and micro-sized fibrous structure, with fiber diameters showing 2 distinct peaks at approximately 300 and 1,300 nm. EDS analysis confirmed the presence of carbon, oxygen, calcium, and silicon, verifying CS incorporation into the PCL matrix. Upon hydration, SEM images revealed micro-flower-shaped CS crystallization adhering to the fibers as discrete or aggregated structures measuring less than 10 μm in diameter (Fig. 1A). Tensile strength and elastic modulus were not significantly affected by CS incorporation or hydration (Fig. 1B and C), indicating that the composite maintained mechanical integrity suitable for clinical handling. In contrast, CS incorporation significantly reduced the water contact angle compared to PCL alone, and hydration further decreased it (Fig. 1D). This increase in hydrophilicity is consistent with previous reports that the incorporation of inorganic materials into organic matrices enhances surface wettability due to improved distribution of inorganic components at the fiber surface [32,33], and suggests altered cellular interactions in biological microenvironments. These physicochemical characteristics align with previously reported electrospun PCL/CS composite scaffolds [36].

Qualitative XRD analysis revealed distinct crystallization behaviors between CS and PCL/CS (Fig. 1E and F). CS powder exhibited characteristic peaks for tricalcium silicate (Ca_3_SiO_5_, alite), dicalcium silicate (β-Ca_2_SiO_4_, belite), and bismuth oxide (Bi_2_O_3_) [9,10]. Upon hydration, a peak corresponding to calcium hydroxide (Ca(OH)_2_) emerged in CS, which diminished with further water immersion, reflecting the release of calcium and hydroxyl ions. In contrast, hydrated PCL/CS did not exhibit the characteristic Ca(OH)_2_ peak; instead, peaks corresponding to CaCO_3_ appeared, accompanied by a decrease in overall crystallinity. These findings indicate that the hydrophobic PCL matrix physically restricts water access to embedded CS particles, altering the hydration pathway. Unlike CS, which retains Ca(OH)_2_ as the primary source of the sustained alkaline burst, the PCL/CS composite did not exhibit detectable Ca(OH)_2_ and instead formed CaCO_3_, likely through carbonation of any transiently generated Ca(OH)_2_ under the restricted hydration conditions imposed by the PCL matrix [9,10]. This altered crystallization pathway has direct implications for the ion release behavior described below.

### Ion release profiles

**Fig. 2.**
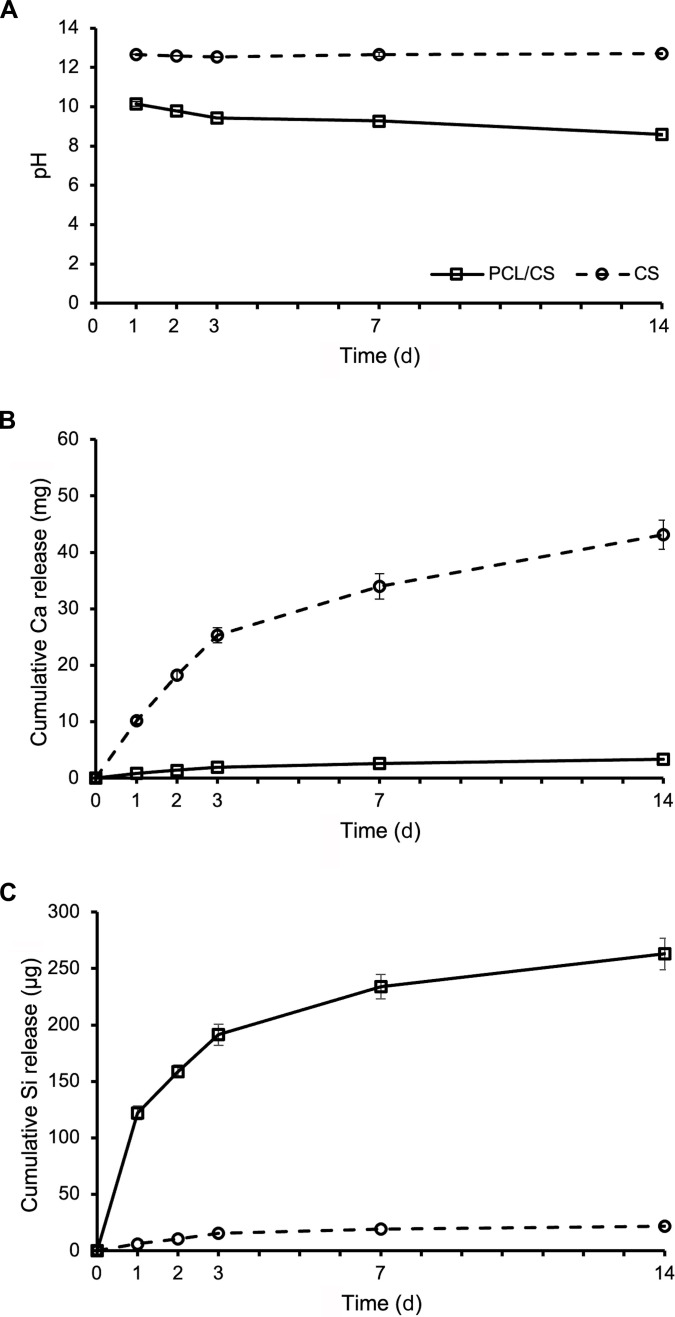
Ion release profiles of calcium silicate (CS) and poly-ε-caprolactone (PCL)/CS. (A) pH of eluates collected from CS (2 g of powder) and PCL/CS (15-cm^2^ membrane) immersed in 10 ml of phosphate-buffered saline (PBS) at the indicated time points. (B and C) Cumulative calcium (Ca) and silicon (Si) release from the same amounts of CS and PCL/CS immersed in 10 ml of double-distilled water (DDW), determined by inductively coupled plasma optical emission spectroscopy. DDW was used to match the conditions for preparing eluates in cell culture experiments. The immersion medium was collected and replaced at 1, 2, 3, 7, and 14 d, and interval-specific values were sequentially summed to yield cumulative release profiles. The amount of each material immersed was scaled to maintain the proportion of each material applied per treatment in the rat pulp–capping model. Data are presented as mean ± SD (*n* = 3). All PCL/CS values were significantly different from CS (*P* < 0.05).

The cytotoxicity of CS during its initial setting phase has been attributed to high alkalinity and elevated calcium ion levels [3,4]. To evaluate changes in ion release from CS and PCL/CS, materials were immersed and eluates were collected at the indicated time points. As shown in Fig. 2A, the pH of CS eluates reached approximately 12.3 within the first day, consistent with previous reports [9,10]. In contrast, PCL/CS exhibited a significantly lower pH of approximately 9.0. Notably, the pH of PCL/CS eluates gradually decreased toward physiological levels over time. This decline can be attributed to the altered crystallization pathway identified by the XRD analysis (Fig. 1E and F). In CS, Ca(OH)_2_ accumulates as a stable hydration product and serves as a sustained alkaline reservoir that maintains the high pH. In contrast, in PCL/CS, the restricted water access within the PCL matrix suppresses bulk Ca(OH)_2_ accumulation; any Ca(OH)_2_ transiently generated is rapidly consumed through carbonation to form CaCO_3_ [9,10], a reaction that concurrently removes hydroxyl ions from the local environment and accounts for the progressive pH reduction toward physiological levels.

Cumulative calcium and silicon ion release was monitored over 14 d (Fig. 2B and C). CS released calcium ions at levels approximately 3 orders of magnitude higher than PCL/CS during the initial phase, consistent with the pronounced burst release driven by rapid Ca(OH)_2_ dissociation. In contrast, PCL/CS exhibited a lower, more sustained calcium release profile. Conversely, silicon ions were more prominently released from PCL/CS, likely reflecting the gradual dissolution of silicate phases from the polymer-encapsulated CS particles. These cumulative release profiles indicate that PCL/CS enables the continuous release of calcium and silicon ions at effective concentrations over an extended period, in contrast to the abrupt, supraphysiological release observed with CS alone.

This controlled ion release pattern aligns with the distinct crystallization behaviors upon hydration (Fig. 1E and F). Importantly, the supraphysiological calcium concentration (approximately 25 mM) and extreme alkalinity (pH ~ 12.3) generated by CS during the initial setting phase, in contrast to the substantially lower levels from PCL/CS (approximately 0.3 mM Ca^2+^, pH ~ 9.0), not merely are cytotoxic to adjacent pulp cells [3,4] but also have important implications for innate immune responses, as CS-induced cell death depletes the local progenitor pool and releases intracellular contents that act as DAMPs, further amplifying the inflammatory response [12]. Whether the ion concentrations released from these materials are sufficient to trigger specific immune cell responses was directly tested in the subsequent in vitro experiments.

### In vitro cytotoxicity and NETosis

**Fig. 3.**
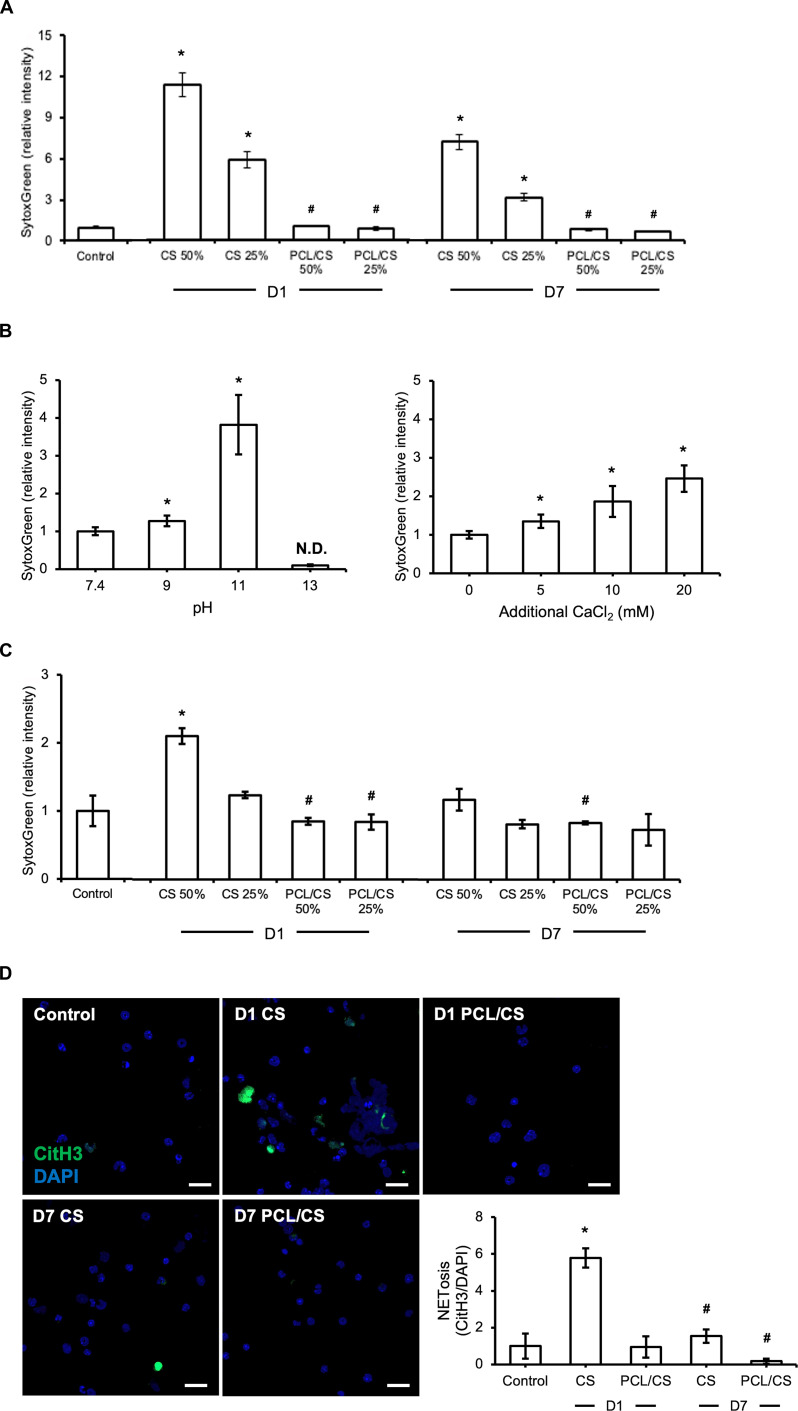

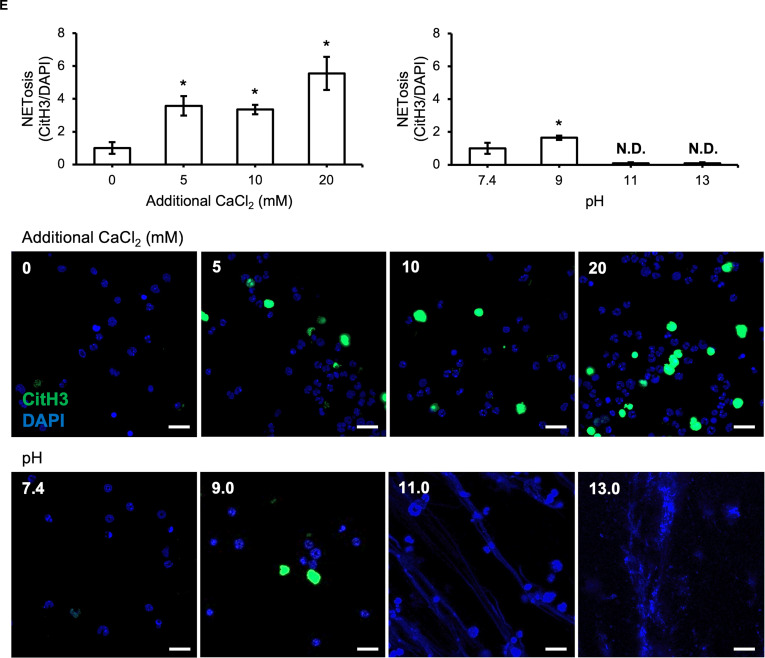
In vitro cytotoxicity and NETosis assessment. Cell death was assessed by SytoxGreen staining, and NETosis was evaluated by citrullinated histone H3 (CitH3) immunostaining; the CitH3-positive area was normalized to the 4′,6-diamidino-2-phenylindole (DAPI)-stained area and expressed as a percentage. (A) SytoxGreen cytotoxicity of primary neutrophils treated with calcium silicate (CS) and poly-ε-caprolactone (PCL)/CS eluate-containing media (25% or 50% v/v in medium). Powdered Dulbecco’s modified Eagle medium (DMEM) was reconstituted with the double-distilled water (DDW)-based eluate (CS: 2 g of powder per 10 ml; PCL/CS: 15-cm^2^ membrane per 10 ml) and applied at the designated concentration. (B) Cytotoxicity of primary neutrophils at varied pH (9, 11, and 13) and culture medium supplemented with additional CaCl_2_ (5, 10, or 20 mM). (C) Cytotoxicity of MDPC-23 pre-odontoblasts treated with CS and PCL/CS eluate-containing media under the same conditions as (A). (D) NETosis quantification of primary neutrophils treated with CS and PCL/CS eluate-containing media (50% v/v) (scale bar = 20 μm). (E) NETosis quantification of primary neutrophils at varied pH and additional CaCl_2_ concentrations (scale bar = 20 μm). N.D., not determined due to alkaline-induced fluorophore degradation at pH ≥ 11. Primary cells were isolated from 3 independent animals, and experiments were independently repeated for each animal. Data are presented as mean ± SD (*n* = 6). *P < 0.05 versus control; ^#^*P* < 0.05 versus CS.

CS is widely recognized for its capacity to promote odontogenic and osteogenic differentiation once the initial hydration phase has subsided, which underlies its status as a gold standard material for vital pulp therapy [5–8]. However, this regenerative potential can be compromised by the acute cytotoxicity and aberrant immune responses provoked by the abrupt ion release during the early hydration stage. Having established that the most pronounced differences in ion release between CS and PCL/CS are concentrated within the initial 24 h (Fig. 2), we next investigated whether these physicochemical differences translate into differential cellular responses using phase-specific eluates (Fig. 3). Eluates were collected during the first 24 h (D1), capturing the initial burst release, and during days 6 to 7 (D7), representing the stabilized late-phase release, and were reconstituted in culture medium at 25% or 50% (v/v), thereby providing graded calcium and pH exposures that bracket the dose–response conditions tested below.

In primary neutrophils, cell death assessed by SytoxGreen fluorescence intensity was markedly increased with the D1 CS 50% eluate, while D7 CS eluates showed attenuated but still detectable cytotoxicity, indicating that the initial burst release is the primary driver (Fig. 3A). PCL/CS eluates maintained cell death at or below control levels across all conditions. MDPC-23 pre-odontoblasts were less susceptible, with only D1 CS 50% producing a modest increase (Fig. 3C), suggesting that neutrophils—the first innate immune cells recruited to the injury site—are more susceptible to the initial ion burst than pre-odontoblasts. Dose–response experiments with independently controlled pH and Ca^2+^ concentrations corroborated the eluate results: elevated pH and Ca^2+^ each increased neutrophil death in a concentration-dependent manner (Fig. 3B). At pH 13, fluorescence was not detectable (N.D.), which is interpreted as a false negative resulting from alkaline DNA denaturation and degradation of the cyanine-based fluorophore [39,40].

In addition to cytotoxicity, the abrupt ion release from CS triggered excessive NETosis in neutrophils. CitH3 immunofluorescence quantification revealed that the D1 CS 50% eluate substantially increased the CitH3^+^/DAPI ratio over control, indicating robust NETosis induction (Fig. 3D). In contrast, the D1 PCL/CS eluate showed NETosis levels comparable to those of the control. These results were corroborated by flow cytometric analysis of SytoxGreen-stained neutrophils treated with D1 eluates (50% v/v), in which CS eluate-treated cells exhibited a fluorescence shift pattern similar to that of the calcium ionophore A23187 positive control, a well-established NETosis inducer, whereas PCL/CS eluate-treated cells remained comparable to untreated controls (Fig. S4). Importantly, the D7 CS eluate exhibited markedly reduced NETosis, returning to near-control levels, directly demonstrating that CS-induced NETosis is primarily attributable to the initial burst release rather than the sustained ion release at later time points. This temporal pattern parallels the cytotoxicity data and reinforces the conclusion that the early physicochemical insult from CS is the critical event that initiates the pathological immune cascade. In the Ca^2+^ and pH dose–response NETosis experiments, Ca^2+^ supplementation significantly increased CitH3 positivity at all concentrations tested (Fig. 3E), consistent with previous reports of calcium-influx-dependent NETosis induction [14–16] and alkaline pH-mediated enhancement of histone citrullination [17,18]. Note that at pH ≥ 11, CitH3 immunostaining was not determined (N.D.) due to alkaline-induced fluorophore degradation, unlike the cytotoxicity assay (Fig. 3B) where pH 11 data were obtainable because of the shorter incubation time and different detection conditions. When correlated with the ion release data (Fig. 2), the initial pH (~12.3) and supraphysiological Ca^2+^ concentration (approximately 25 mM) of CS D1 eluates clearly exceed both the cytotoxicity and NETosis thresholds established in the dose–response experiments. Although pH 9 alone was sufficient to induce a modest increase in NETosis in the dose–response assay (Fig. 3E), the dose–response experiments employed a defined pH maintained throughout the culture period, whereas eluate-based experiments involved dilution into culture medium, in which the buffering capacity of the medium substantially attenuates the pH. These differences in experimental conditions account for the absence of NETosis observed with PCL/CS eluates.

Collectively, while the regenerative capacity of CS is well established in the literature [5–8], the present results identify the abrupt ion release during the initial hydration phase as a major contributor to direct cytotoxicity and excessive NETosis in neutrophils. The marked reduction in both cytotoxicity and NETosis-inducing capacity of CS eluates at D7 suggests that once the initial burst subsides, CS can exert its inherent bioactive properties. However, cytotoxicity and NETosis represent mechanistically distinct consequences of this early burst. Cytotoxicity causes membrane-compromising cell death—evidenced by SytoxGreen uptake—that depletes the local progenitor pool and releases intracellular DAMPs [3,4,12]. NETosis, by contrast, is an active, regulated form of neutrophil death that releases extracellular traps decorated with granule proteins such as NE and histones—components that function as secondary DAMPs capable of directly skewing macrophage polarization toward a pro-inflammatory M1 phenotype [19]. Thus, the pathological impact of the CS burst extends beyond simple cell loss: by simultaneously triggering excessive NETosis, it actively programs the subsequent immune microenvironment toward sustained inflammation, a consequence that cytotoxicity alone would not predict. PCL/CS provides a strategy to circumvent both of these pathological cascades triggered by the initial burst while preserving the regenerative inductive capacity of CS.

### In vitro macrophage polarization

**Fig. 4.**
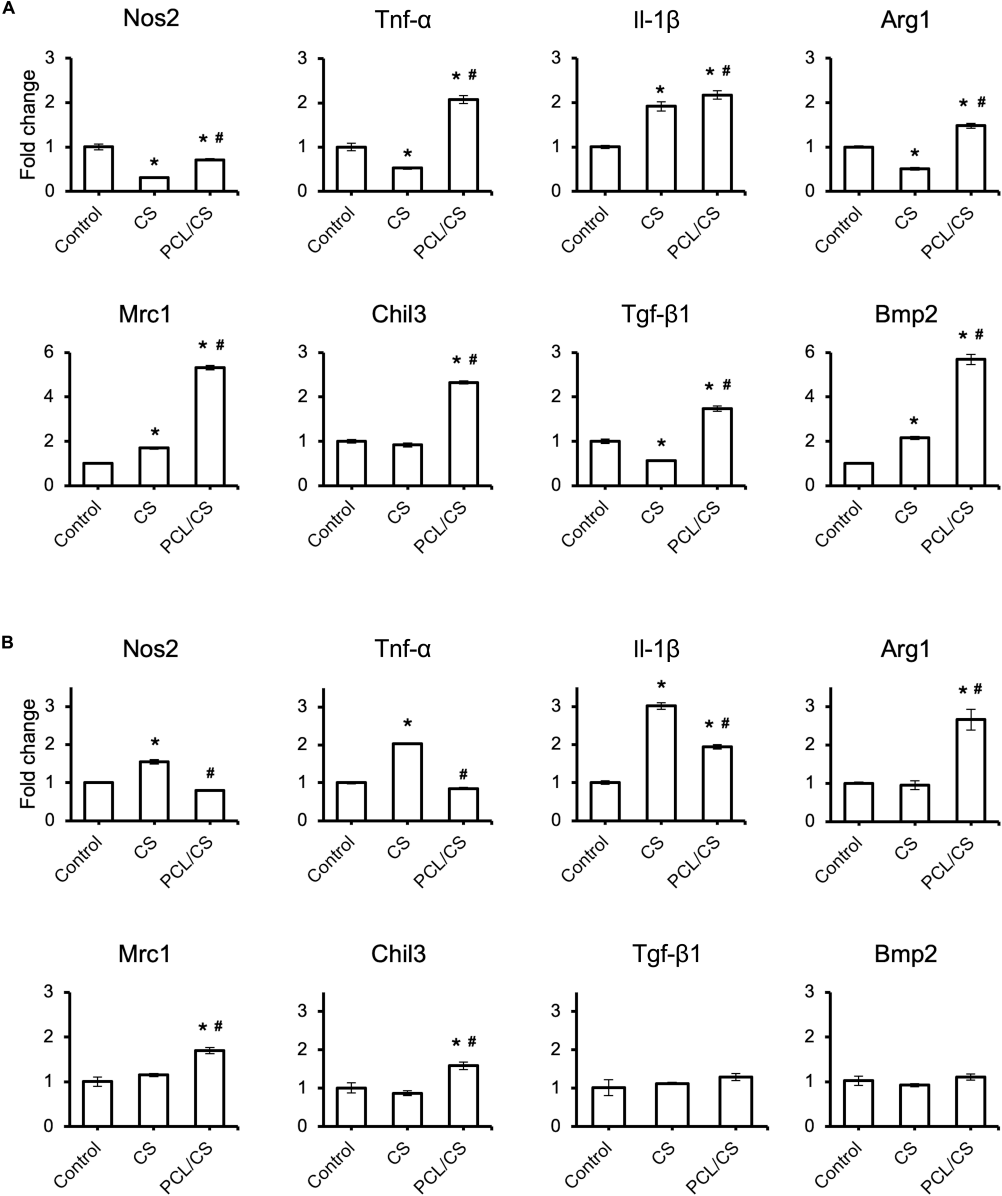
In vitro macrophage polarization by calcium silicate (CS) and poly-ε-caprolactone (PCL)/CS eluates and neutrophil extracellular traps (NETs). (A) Quantitative real-time polymerase chain reaction (RT-qPCR) analysis of M1 (Nos2, Tnf-α, and Il-1β) and M2 (Arg1, Mrc1, and Chil3) marker expression in macrophages treated with CS or PCL/CS eluates (25% v/v). (B) RT-qPCR analysis of M1 and M2 marker expression in macrophages treated with NETs isolated from CS or PCL/CS eluate-stimulated neutrophils. Primary cells were isolated from 3 independent animals, and experiments were independently repeated for each animal (*n* = 3 biological replicates). RT-qPCR was performed in technical triplicate. Data are presented as mean ± SD (*n* = 3). *P < 0.05 versus control; ^#^*P* < 0.05 versus CS.

Having established that the abrupt ion release from CS triggers excessive NETosis, generating NET components that can function as secondary DAMPs, we next investigated whether the material eluates and NET-derived products differentially influence the macrophage phenotype. Primary macrophages were treated with D1 CS or PCL/CS eluates (25% v/v) or with NETs isolated from D1 CS- or PCL/CS eluate-stimulated neutrophils, and the expression of M1 markers (Nos2, Tnf-α, and Il-1β), M2 markers (Arg1, Mrc1, and Chil3), and proregenerative mediators (Tgf-β1 and Bmp2) was assessed by RT-qPCR (Fig. 4).

When macrophages were directly treated with material eluates (Fig. 4A), the CS eluate suppressed Nos2 and Tnf-α while upregulating Il-1β and, paradoxically, the M2-associated marker Mrc1—albeit to a significantly lower level than the PCL/CS eluate—a pattern that does not conform to classical M1 polarization but rather suggests dysregulated macrophage activation. In contrast, the PCL/CS eluate markedly upregulated the M2 markers Arg1, Mrc1, and Chil3 as well as the proregenerative mediators Tgf-β1 and Bmp2. The PCL/CS eluate also increased Tnf-α and Il-1β expression; however, the overall expression profile—dominated by robust M2 marker and proregenerative mediator upregulation—indicates a predominantly anti-inflammatory and pro-healing macrophage phenotype.

To determine whether the differential NETosis levels between CS and PCL/CS translate into macrophage polarization through NET-derived components, macrophages were treated with NETs isolated from D1 CS- or PCL/CS eluate-stimulated neutrophils (Fig. 4B). CS-derived NETs upregulated all M1 markers tested (Nos2, Tnf-α, and Il-1β), providing direct experimental evidence that NET components generated during excessive NETosis are sufficient to polarize macrophages toward a pro-inflammatory phenotype [19]. PCL/CS-derived NETs—consistent with the minimal NETosis observed in the PCL/CS group (Fig. 3D)—did not significantly increase Nos2 or Tnf-α expression above control levels. These results establish the mechanistic link between NETosis and macrophage polarization that constitutes the NETosis–macrophage axis central to this study: excessive NETosis triggered by CS actively drives M1 polarization, whereas the attenuated NETosis in the PCL/CS group permits M2-dominant macrophage programming.

Taken together, these results reveal that the macrophage polarization in this system is governed by 2 complementary pathways: (a) direct eluate effects, in which the attenuated ion release from PCL/CS promotes an M2/proregenerative macrophage phenotype, while the CS eluate dysregulates macrophage function, and (b) NET-mediated effects, in which CS-derived NET components actively skew macrophages toward a pro-inflammatory M1 phenotype. The convergence of these 2 pathways in the PCL/CS group—reduced pro-inflammatory stimulation through attenuated NETosis combined with direct promotion of M2 polarization—supports a favorable immunomodulatory microenvironment. The upregulation of Tgf-β1 and Bmp2 in the PCL/CS eluate group is particularly noteworthy, as these mediators are known to promote odontoblast differentiation and dentin matrix secretion [21,22], providing a mechanistic link between macrophage polarization and the regenerative outcomes examined below.

### In vitro odontoblastic differentiation

**Fig. 5.**
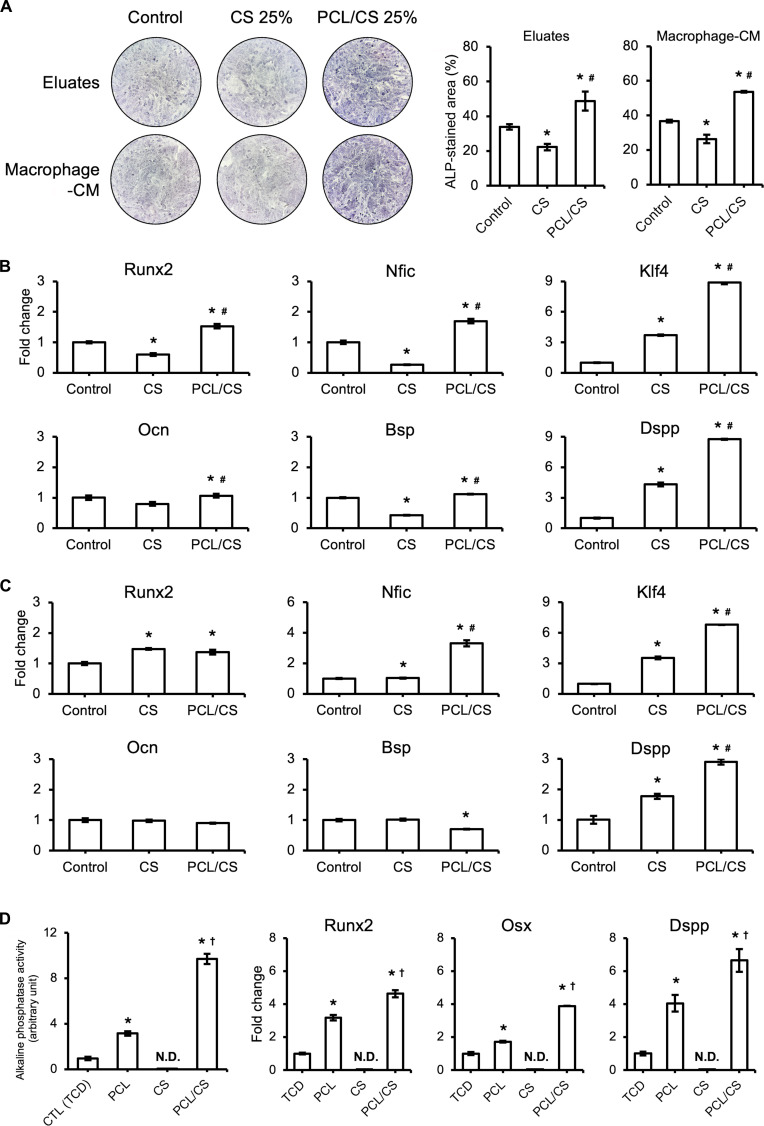
In vitro odontoblastic differentiation of MDPC-23 cells. (A) Alkaline phosphatase (ALP) staining in MDPC-23 cells cultured with calcium silicate (CS) or poly-ε-caprolactone (PCL)/CS eluate-containing media (25% v/v in differentiation medium) and with macrophage-conditioned media (CM) (1:5 dilution in differentiation medium). (B) Quantitative real-time polymerase chain reaction (RT-qPCR) analysis in MDPC-23 cells cultured with CS or PCL/CS eluate-containing media. (C) RT-qPCR analysis in MDPC-23 cells cultured with macrophage-CM. (D) ALP activity and RT-qPCR analysis in MDPC-23 cells cultured on tissue culture dishes (TCD), PCL, CS, and PCL/CS for 7 d. N.D., not determined. Data are presented as mean ± SD (*n* = 4 for ALP activity; *n* = 3 for RT-qPCR). *P < 0.05 versus control; ^#^*P* < 0.05 versus CS; ^†^*P* < 0.05 versus PCL.

Having established the immunomodulatory cascade from ion release through NETosis to macrophage polarization, we next examined the functional consequences for odontoblastic differentiation. MDPC-23 pre-odontoblasts were cultured with CS or PCL/CS eluates (25% v/v in differentiation medium), and differentiation was assessed by ALP staining and RT-qPCR for odontogenic markers (Fig. 5A and B). ALP staining revealed that the PCL/CS eluate markedly enhanced ALP-stained area compared to both control and CS eluate conditions (Fig. 5A). RT-qPCR analysis showed that the CS eluate broadly suppressed odontogenic marker expression, including Runx2, Nfic, Ocn, and Bsp; notably, however, the CS eluate increased Klf4 and Dspp expression above control levels. In contrast, the PCL/CS eluate upregulated all markers tested, with particularly pronounced increases in Klf4 and Dspp (Fig. 5B). The robust upregulation of Klf4 and Dspp by the PCL/CS eluates is noteworthy, as this expression profile is consistent with the transcription factor signature associated with odontoblast terminal differentiation and physiological dentinogenesis reported in previous studies [41–43]. In particular, Klf4 has been shown to be specifically expressed in polarizing odontoblasts [43] and to promote dentinogenesis through the Nfic–Klf4–Dmp1–Dspp pathway [41,42], a pathway whose upstream component Nfic was also markedly upregulated by the PCL/CS eluate (Fig. 5B). These results indicate that the tailored ion release from PCL/CS promotes an odontoblast-associated differentiation program that is not achieved by CS eluates.

To determine whether the macrophage polarization state induced by the materials functionally translates into differential odontoblastic differentiation, MDPC-23 cells were cultured with CM from macrophages previously treated with CS or PCL/CS eluates (Fig. 5A and C). ALP staining demonstrated that PCL/CS macrophage-CM promoted greater ALP-stained area than CS macrophage-CM (Fig. 5A). RT-qPCR revealed that PCL/CS macrophage-CM markedly upregulated Nfic, Klf4, and Dspp, whereas CS macrophage-CM showed significant but lower increases in these markers compared to PCL/CS macrophage-CM (Fig. 5C). Runx2 expression was significantly elevated in both groups relative to that in the control, with no significant difference between CS and PCL/CS macrophage-CM. It should be noted that the control for macrophage-CM experiments is CM from untreated macrophages, which already contains constitutively secreted cytokines and growth factors; this elevated baseline accounts for the relatively smaller differences compared to the eluate experiments, yet PCL/CS macrophage-CM still produced substantial upregulation of Klf4 and Dspp. The observation that PCL/CS macrophage-CM recapitulated the Nfic, Klf4, and Dspp upregulation pattern seen with direct eluate treatment (Fig. 5B) indicates that CM from M2-polarized macrophages is sufficient to promote odontoblastic differentiation, establishing a functional link between immune modulation and regenerative outcome [21,22]. These findings support the concept that the immunomodulatory microenvironment created by PCL/CS—through attenuated NETosis and consequent M2 macrophage polarization—may contribute to dentin–pulp regeneration, a mechanism that extends beyond the direct material–cell interactions conventionally attributed to CS-based biomaterials.

Odontoblastic differentiation was also assessed under direct material substrate conditions (Fig. 5D). MDPC-23 cells were cultured on TCD, PCL, CS, and PCL/CS for 7 d, and ALP activity and odontogenic marker expression were evaluated. PCL/CS supported the highest ALP activity and upregulated Runx2, Osx, and Dspp expression compared to both TCD and PCL alone, confirming that the combination of nanofibrous topography and sustained ion release synergistically promotes odontoblastic differentiation. PCL fibers alone also enhanced differentiation relative to TCD, consistent with previous reports on topography-mediated odontoblast induction [28–30]. Notably, differentiation data for the CS substrate group could not be determined (N.D.) because CS-induced cytotoxicity (Fig. 3) prevented cell survival sufficient for differentiation analysis.

### In vivo early host response

**Fig. 6.**
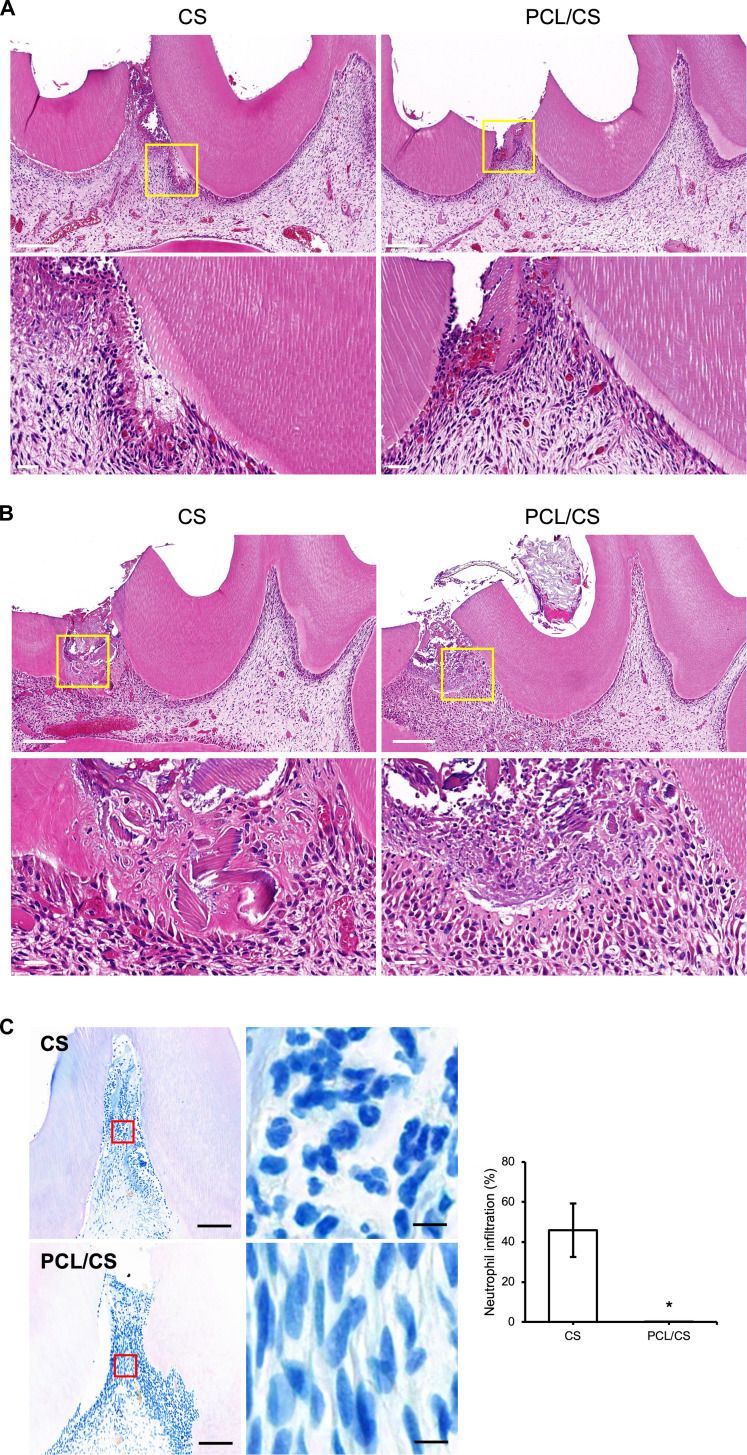
In vivo early host response to calcium silicate (CS) and poly-ε-caprolactone (PCL)/CS. Representative hematoxylin and eosin (H&E)-stained microscopic images of rat first molars with exposed pulps treated with CS (left) and PCL/CS (right) at 2 d (A) and 7 d (B) post-op. Boxes in micrographs (scale bar = 100 μm) in the upper panel show the enlarged images in the lower panel (scale bar = 20 μm). (C) Giemsa-stained images at day 7 post-op. Boxes (scale bar = 100 μm) show the enlarged images (scale bar = 10 μm). For quantitative analysis, 3 regions of interest (ROIs) of identical dimensions were positioned within the pulp tissue adjacent to the exposure site in each section. Neutrophil infiltration was expressed as the percentage of neutrophils, identified by their characteristic multilobed nuclei, relative to total cell count within each ROI. Data are presented as mean ± SD (*n* = 3 to 4 animals per group, with 3 ROIs per sample). *P < 0.01 versus CS.

Early host responses to CS and PCL/CS were evaluated in a rat molar pulp exposure model. As shown in Fig. 6A, at 2 d post-op, the CS group exhibited odontoblastic layer detachment from the dentin and extensive infiltration of inflammatory cells, which extended deep into the tissue. In contrast, the PCL/CS group showed no apparent detachment and significantly fewer infiltrating inflammatory cells. At 7 d post-op (Fig. 6B), a substantial number of inflammatory cells remained in the CS group, whereas inflammation appeared to subside in the PCL/CS group. Giemsa staining, which is useful for identifying inflammatory cells including neutrophils, revealed persistent neutrophils in the CS group at 7 d post-op, but not in the PCL/CS group (Fig. 6C). Quantitative analysis confirmed that the CS group exhibited significantly higher neutrophil infiltration compared to the PCL/CS group (Fig. 6C). These findings are consistent with the in vitro observations (Fig. 3), where CS eluates induced significant cytotoxicity and NETosis at concentrations and pH levels that exceeded the thresholds identified in the dose–response experiments. The extensive inflammatory cell infiltration and odontoblastic layer detachment observed in the CS group likely reflect the combined effects of direct cytotoxicity [3,4,12] and the subsequent innate immune cascade initiated by these stimuli.

**Fig. 7.**
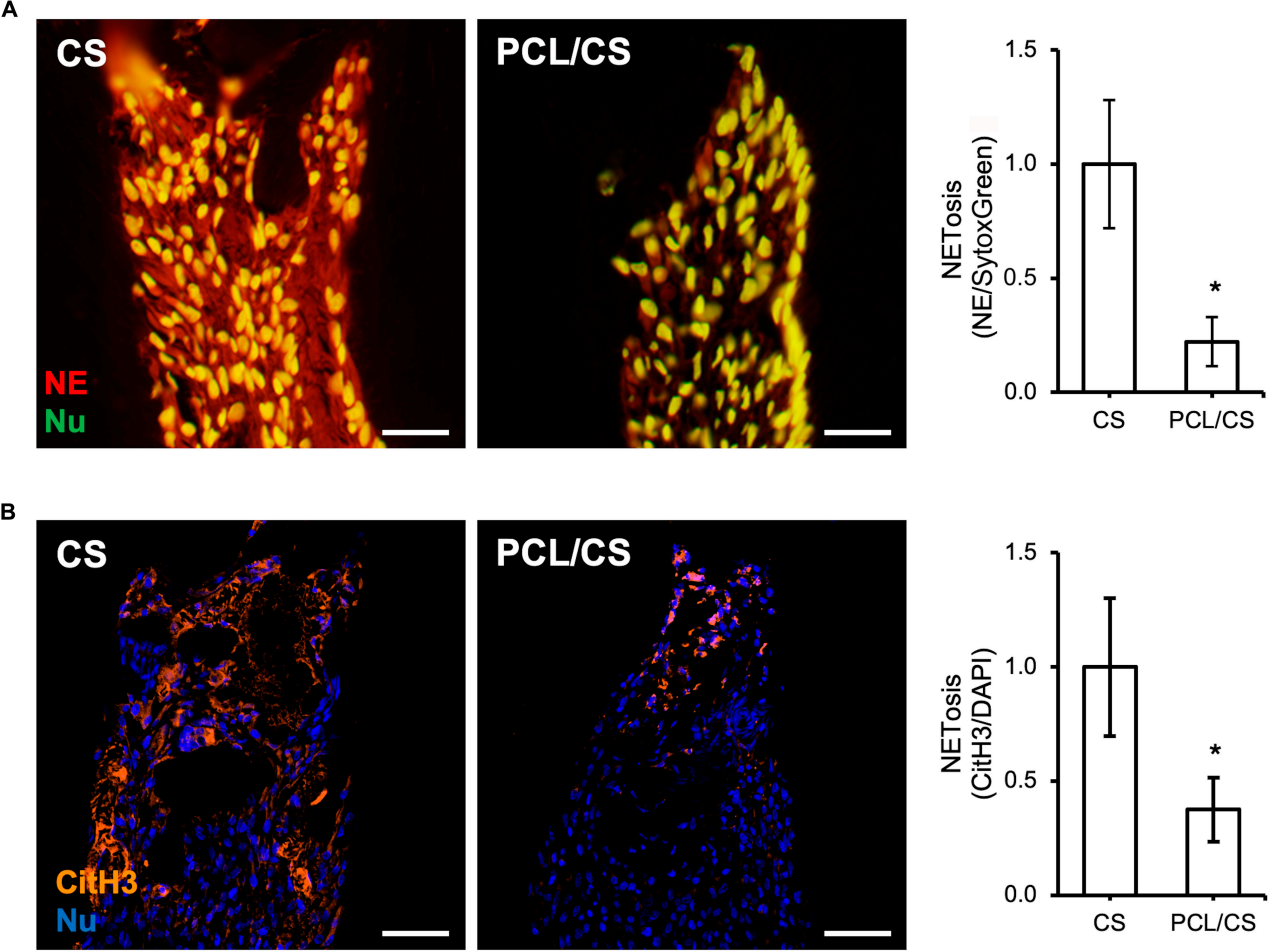
In vivo NETosis assessment. (A) Neutrophil elastase (NE) and (B) citrullinated histone H3 (CitH3) immunostaining in the pulp horn regions of rat first molars treated with calcium silicate (CS) (left) and poly-ε-caprolactone (PCL)/CS (right) at 7 d post-op (scale bar = 50 μm). For quantitative analysis, 3 regions of interest (ROIs) of identical dimensions were randomly positioned within the pulp horn area adjacent to the exposure site in each section. Image-based quantification was performed using the ImageJ software with a uniform threshold applied across all images. The positively stained area for each marker was normalized to the corresponding nuclear-stained area (SytoxGreen for NE; 4′,6-diamidino-2-phenylindole [DAPI] for CitH3) within the same ROI. Data are presented as mean ± SD (*n* = 3 to 4 animals per group, with 3 ROIs per sample). *P < 0.05 versus CS.

NE and CitH3, markers of NETosis, were immunostained to assess NETosis levels at 7 d post-op (Fig. 7). In the CS group, the exposed pulp horns showed extensive NE and CitH3 staining, indicating excessive NETosis. In contrast, the NE- and CitH3-positive area ratios were significantly lower in the PCL/CS group. These in vivo NETosis findings corroborate the in vitro CitH3 immunostaining and flow cytometric analysis data (Fig. 3 and Fig. S4), confirming that the attenuated ion release from PCL/CS is sufficient to reduce NETosis not only in isolated neutrophil cultures but also in the complex tissue microenvironment.

**Fig. 8.**
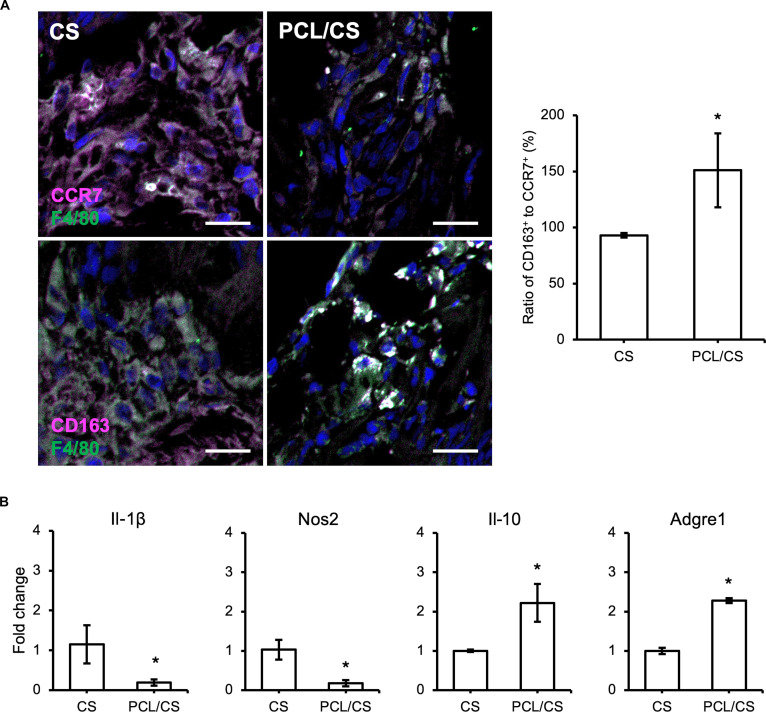
In vivo macrophage polarization. (A) CCR7 (upper) and CD163 (lower) immunostaining with F4/80 (Adgre1) costaining in the pulp horn regions of rat first molars treated with calcium silicate (CS) (left) and poly-ε-caprolactone (PCL)/CS (right) at 7 d post-op (scale bar = 20 μm). For quantitative analysis, 3 regions of interest (ROIs) of identical dimensions were randomly positioned within the pulp horn area adjacent to the exposure site in each section. CCR7–F4/80 and CD163–F4/80 double-positive cells were counted within each ROI, and the CD163/CCR7 ratio was calculated. (B) Quantitative real-time polymerase chain reaction (RT-qPCR) analysis of Il-1β, Nos2, Il-10, and Adgre1 expression in coronal pulp tissues at 7 d post-op. Data are presented as mean ± SD (*n* = 3 to 4 animals per group; 3 ROIs per sample for immunohistochemistry (IHC); RT-qPCR performed in technical triplicate). *P < 0.05 versus CS.

To further assess whether the differential NETosis levels translated into macrophage polarization in vivo, CCR7 (M1) and CD163 (M2) were immunostained on tissue sections at 7 d post-op (Fig. 8A). F4/80 (Adgre1), a macrophage-specific marker absent in granulocytes, was used to confirm macrophage identity. Consistent with the NETosis results, the CS group exhibited intense CCR7 staining with relatively less CD163 staining, whereas the PCL/CS group prominently displayed high CD163 positivity. Quantitative analysis of CCR7–F4/80 and CD163–F4/80 double-positive cells confirmed a significantly higher CD163/CCR7 ratio in the PCL/CS group (Fig. 8A), indicating that attenuated NETosis in the PCL/CS group facilitated M2 polarization, while the CS group sustained M1-dominant polarization. Gene expression analysis of coronal pulp tissues at 7 d post-op further supported these findings (Fig. 8B): the M1 markers Il-1β and Nos2 were expressed at significantly higher levels in the CS group, whereas the M2 marker Il-10 and the macrophage marker Adgre1 were significantly elevated in the PCL/CS group. The higher Adgre1 expression in the PCL/CS group suggests that the transition from neutrophil-dominant to macrophage-dominant infiltration occurred more rapidly than in the CS group, where persistent NETosis delayed this transition.

Taken together, these in vivo findings validate the NETosis–macrophage axis identified in our in vitro experiments (Figs. 3 and 4): excessive NETosis triggered by CS actively drives M1-dominant macrophage polarization and prolongs inflammation, whereas the attenuated NETosis in the PCL/CS group permits timely M1-to-M2 transition, consistent with an immunomodulatory microenvironment supportive of tissue regeneration.

### Dentin–pulp complex regeneration

**Fig. 9.**
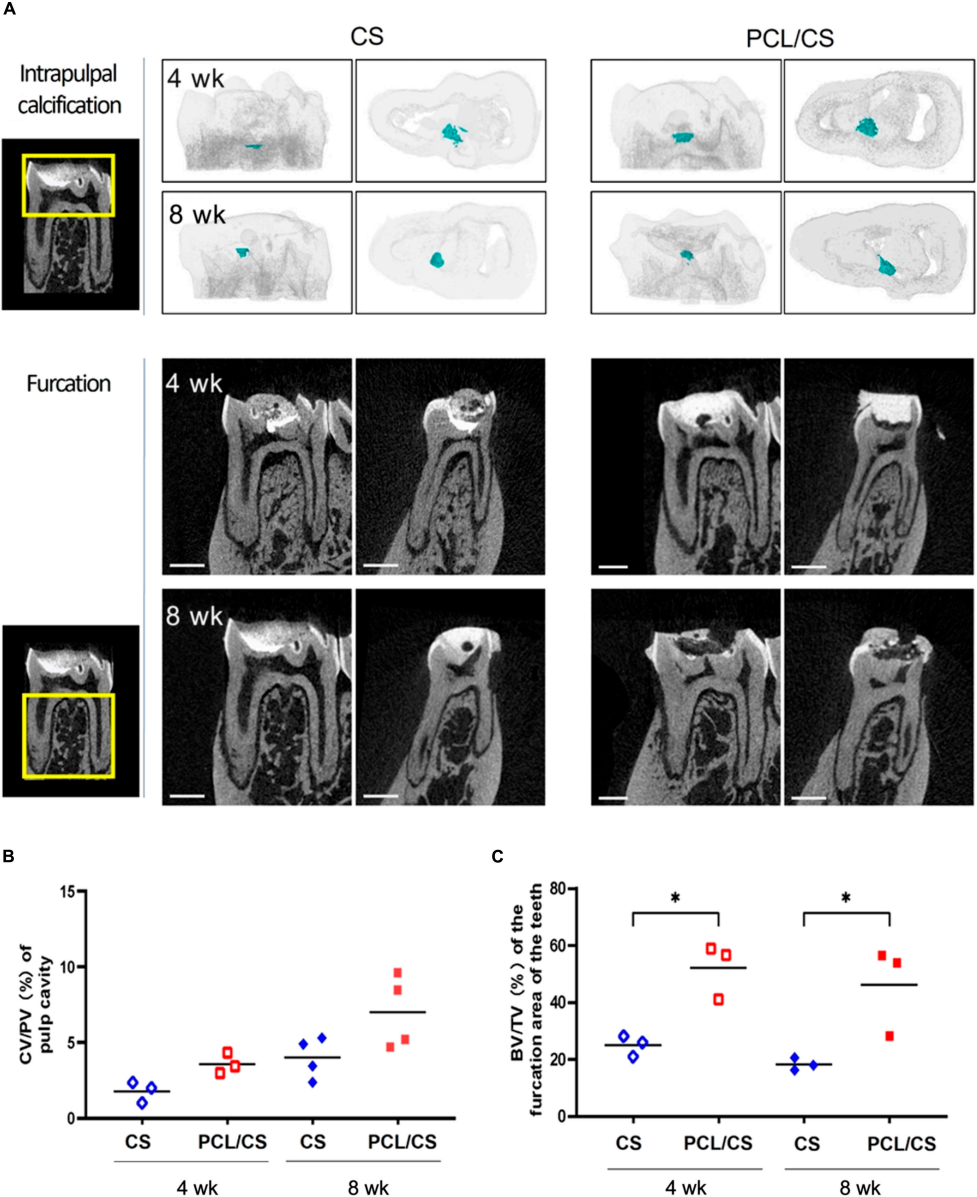
Micro-computed tomography (micro-CT) evaluation of calcium silicate (CS) and poly-ε-caprolactone (PCL)/CS regenerative outcomes. (A) Reconstructed micro-CT images of rat first molars treated with CS (left) and PCL/CS (right) at 4 and 8 weeks post-op. Sagittal (mesiodistal) and horizontal sectional images are presented (scale bar = 1 mm). (B) Volume percentage of calcified tissue within pulp chamber in the CS and PCL/CS groups. (C) Bone volume fraction (BV/TV) at the furcal area in the CS and PCL/CS groups. Data are presented as mean ± SD (*n* = 3 to 4 per group). *P < 0.05 versus CS.

Micro-CT analysis was performed to evaluate barrier formation, intrapulpal calcification, and the integrity of bone tissue around the furcation area. In the horizontal sections of the PCL/CS group, a thick, continuous calcified barrier was observed beneath the pulp-exposed area, extending along with the adjacent preexisting dentin (Fig. 9A). In contrast, the CS group displayed thinner barriers and diffuse calcified opaque material in the pulp chamber, resulting in no significant difference between the 2 groups in the percentage of calcified tissue volume within the pulp chamber (Fig. 9B). Thus, although the 2 groups showed comparable calcified tissue volume by micro-CT quantification, they differed mainly in the organization and continuity of the calcified barrier. Notably, the CS group showed significantly less alveolar bone volume ratio in the furcation area at both 4 and 8 weeks post-op (Fig. 9C), indicating delayed bone tissue regeneration.

**Fig. 10.**
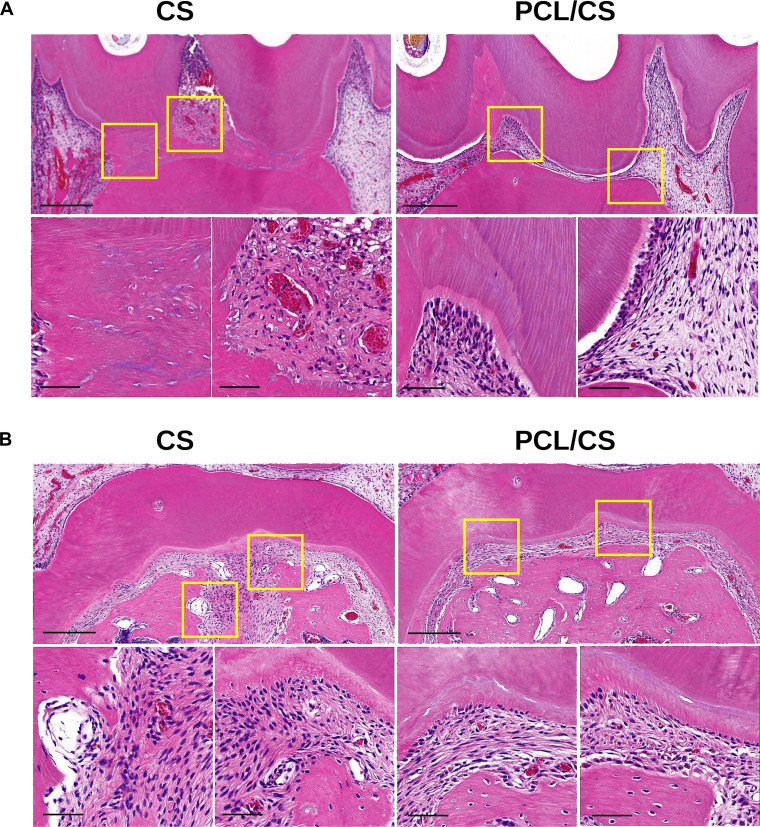
Histological evaluation of calcium silicate (CS) and poly-ε-caprolactone (PCL)/CS regenerative outcomes. Representative microscopic images of rat first molars treated with CS (left) and PCL/CS (right) at 8 weeks post-op in the coronal area (A) and the furcation area (B). Boxes in low-magnification images (upper panel; scale bar = 500 μm) indicate the regions enlarged in the lower panel (scale bar = 20 μm). Hematoxylin and eosin (H&E) staining. Representative images are shown from *n* = 3 to 4 animals per group.

Histological analysis of the coronal and furcation areas was conducted to evaluate calcified barrier formation, the odontoblastic cell layer, and the integrity of pulp and furcal bone tissues. In the coronal area at 8 weeks, calcified barriers beneath the pulp-exposed area and discrete mineralized matrices in the pulp chamber were observed in both groups (Fig. 10A). The calcified barriers in the CS group appeared as an amorphous matrix with cell inclusions and barely observable dentinal tubules, resembling typical osteodentin [33,44]. Notably, the PCL/CS group formed a substantial calcified barrier with tubular dentin-like structures (Fig. 10A). Furthermore, in the PCL/CS group, columnar polarized odontoblast-like cells with long processes were observed. Regarding pulp integrity, the CS group showed features of inflammation and necrosis to some extent, as previously reported [45]. In contrast, the PCL/CS group exhibited markedly reduced inflammatory features, and necrotic changes were scarcely observed. At the furcation area, at 8 weeks, notable inflammation and bone resorption were observed in the CS group (Fig. 10B), which is consistent with the reduced furcal bone volume seen in the micro-CT analyses. In contrast, the PCL/CS group showed reduced signs of inflammation and bone resorption.

The distinct regenerative outcomes between the 2 groups can be interpreted in the context of the NETosis–macrophage axis identified in this study. The CS group, characterized by excessive NETosis and sustained M1 polarization (Figs. 7 and 8), produced features of repair rather than regeneration—amorphous osteodentin with cell inclusions [33,44]—whereas the PCL/CS group, which mitigated NETosis and facilitated M2 polarization, showed more organized dentin formation, characterized by tubular dentin and polarized odontoblasts. Notably, these in vivo histological outcomes are concordant with the in vitro differentiation data (Fig. 5): PCL/CS eluates and M2 macrophage-CM both promoted an odontoblast-associated expression profile, including the marked upregulation of Klf4, Nfic, and Dspp—markers closely linked to physiological dentinogenesis [41–43]—whereas CS conditions failed to elicit this pattern. This concordance between the molecular signatures in vitro and the tissue-level outcome in vivo supports the functional role of the immunomodulatory microenvironment in directing dentin formation [21,22]. This work contributes to linking the tailored ion release kinetics of PCL/CS to NETosis-associated immune programming in the dentin–pulp complex. The contribution of nanofibrous topography to this regenerative outcome should also be considered. PCL fibers have been reported to have a high affinity for calcium ions, promoting their retention and nucleation [46]. We previously demonstrated that PCL fibers in pulp capping procedures facilitate the formation of a thicker dentin bridge [33]. In the present study, the sustained release of calcium ions at low but effective concentrations from PCL/CS (Fig. 2), combined with the nanofibrous topographical cues, likely contributed synergistically to odontoblast differentiation (Fig. 5D). Consistent with this interpretation, nanofibrous topography has been shown to direct odontoblastic differentiation via canonical Wnt signaling [28] and Wnt5a-mediated cellular polarization [29] and more recently to restore cell–cell junctions in odontoblasts through Wnt5a–Cdc42 activation [30].

It should be noted that CS provoked furcal bone defects, while PCL/CS mitigated their occurrence. When intrapulpal inflammation spreads via accessory canals, it affects the interradicular furcation area [47]. Periodontal involvement in the furcation area often presents healing challenges due to the limited vascularization of this region, which restricts the delivery of essential nutrients and reparative cells [48,49]. The reduced alveolar bone resorption observed with PCL/CS was likely due to the decreased transmission of inflammatory byproducts into the furcation area.

Clinically, the sheet form of PCL/CS offers several practical advantages over CS in its powder form. This design facilitates precise application and substantially reduces placement errors. In addition, the PCL/CS sheet eliminates the need for long setting times, enabling immediate clinical use. Regarding postoperative pain, which typically peaks 3 to 5 h after CS application [50], the preset nature of PCL/CS may reduce patient discomfort by avoiding the acute alkaline and cytotoxic insult associated with the initial setting reaction.

Several limitations of this study should be acknowledged. First, although NETs isolated from CS-stimulated neutrophils directly induced M1 macrophage polarization in vitro, the causal contribution of NETosis to the in vivo regenerative outcome remains correlative; its definitive establishment would require loss-of-function approaches, such as PAD4-deficient models. Second, the interplay among ion release, NETosis, and macrophage reprogramming was characterized primarily through correlative in vitro and in vivo observations, and the relative contribution of each pathway to the final regenerative outcome remains to be fully delineated. Third, the rat molar pulp exposure model used healthy pulps and may not fully recapitulate the inflamed pulp microenvironment typically encountered in clinical caries or trauma; interspecies differences in pulp volume, immune kinetics, and dentinogenesis also warrant caution when extrapolating these findings to humans. The immunomodulatory effects of PCL/CS observed in this study provide a foundation for future multi-omic studies, including single-cell transcriptomics and spatial proteomics, to identify specific molecular pathways through which excessive NETosis impairs the M2 transition. Such insights will provide a comprehensive understanding of the therapeutic potential of PCL/CS in regenerative therapeutics.

## Conclusion

In this study, we engineered an electrospun PCL/CS composite fiber that reconfigures the hydration and crystallization kinetics of CS, converting the abrupt burst release of calcium and hydroxyl ions into a sustained, controlled release profile. This tailored ion release mitigated the acute cytotoxicity and excessive NETosis that characterize the early host response to CS, as demonstrated in vitro in primary neutrophils. Mechanistically, the attenuated NETosis in the PCL/CS group consequently reduced M1 macrophage polarization, as CS-derived NETs were shown to directly drive M1 polarization in vitro. In parallel, PCL/CS eluates promoted an anti-inflammatory and proregenerative macrophage phenotype. This convergence of 2 complementary immunomodulatory pathways—reduced pro-inflammatory NET-mediated signaling and direct promotion of M2 polarization—was validated in vivo. The resulting immunomodulatory microenvironment supported improved dentin–pulp regeneration characterized by organized tubular dentin with polarized odontoblasts, consistent with the odontoblastic differentiation profile observed in vitro—in contrast to the amorphous osteodentin typically produced by CS. Furthermore, PCL/CS mitigated furcal bone resorption associated with CS-induced intrapulpal inflammation. These findings indicate that modulating early innate immune responses through tailored ion release via the NETosis–macrophage axis represents an effective strategy for improving regenerative outcomes in dentin–pulp complex tissue engineering.

## Data Availability

The data supporting this study’s findings are available from the corresponding authors on reasonable request.

## Supplementary Materials

Supplementary 1 Graphical Abstract Figs. S1 to S4 Table S1
