# Supplementary material for: Tailored Ion Release of Polycaprolactone and Calcium Silicate Composite Fibers Attenuates Neutrophil Extracellular Trap Formation for Dentin–Pulp Complex Regeneration
Source: Biomater Res. 2026 Jul 14;30:0388. doi: 10.34133/bmr.0388 (PMC13365599; doi:10.34133/bmr.0388)
Supplement: Supplementary 1 — Graphical Abstract Figs. S1 to S4 Table S1 [file bmr.0388.f1.docx]

**GRAPHICAL ABSTRACT**

**
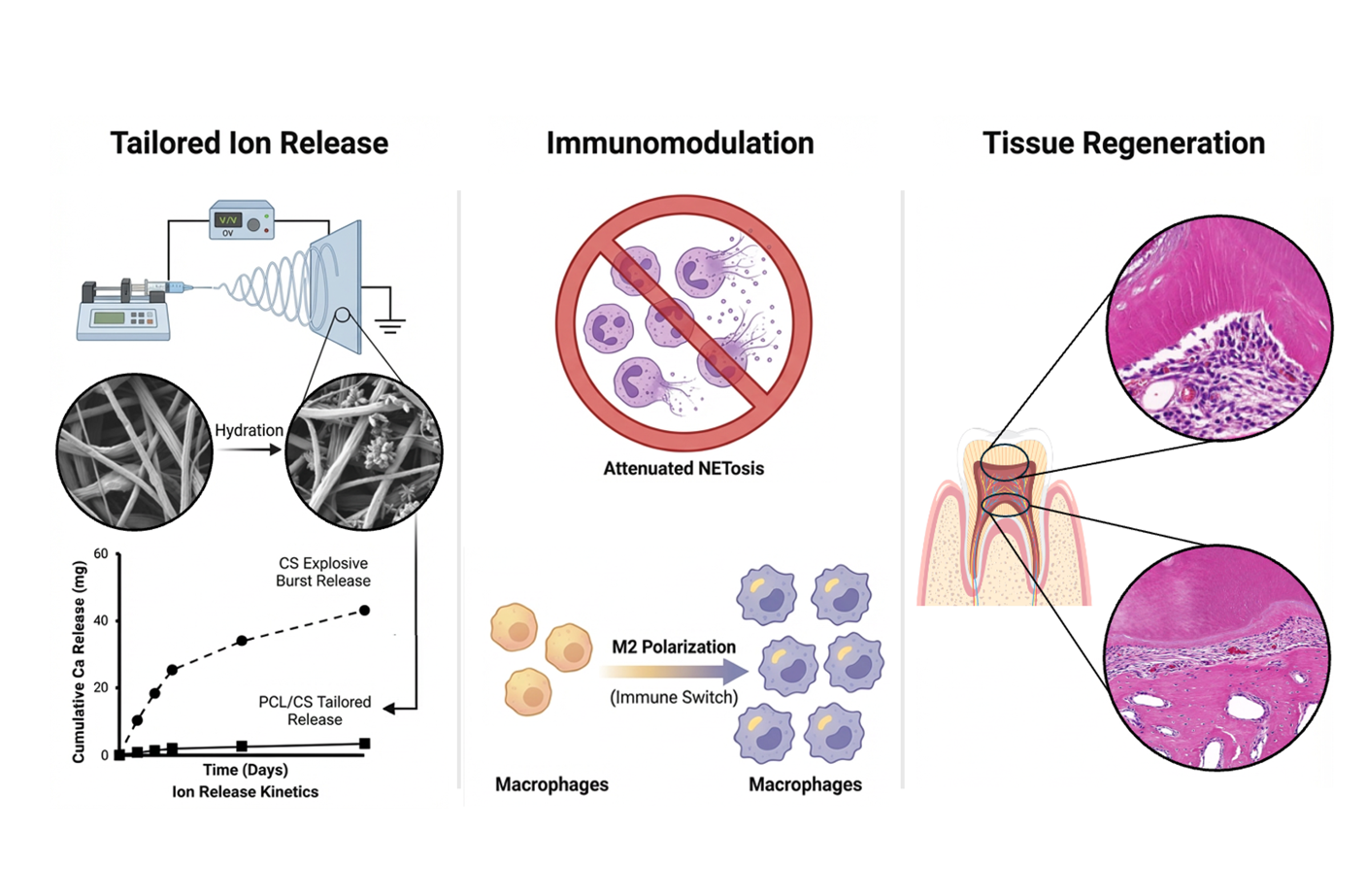
**

Electrospun PCL/CS composite fibers enable tailored ion release that attenuates excessive NETosis. CS-derived NETs drove M1 macrophage polarization, whereas attenuated NETosis in the PCL/CS group permitted M2 polarization, supporting physiological dentin–pulp complex regeneration with tubular dentin formation.

**SUPPLEMENTARY MATERIALS**

**Table S1**. **Sequences of primers used in RT-qPCRs**

| **Rat primers** | | |
| --- | --- | --- |
| Gene | Primer sequence | Accession No. |
| *Gapdh* | F: 5′-CAAGTTCAACGGCACAGTCA-3′  R: 5′-CCCCATTTGATGTTAGCGGG-3′ | NM_017008.4 |
| *Adgre1* | F: 5′-TGCTCCTCTTCTGGGGCTT-3′  R: 5′-AGTGCAGGTGGCATAAGCTG-3′ | NM_001007557.2 |
| *Il1b* | F: 5′-TCATCTTTGAAGAAGAGCCCG-3′  R: 5′-TCAGACAGCACGAGGCATTT-3′ | NM_031512.2 |
| *Il10* | F: 5′-TTCCCTGGGAGAGAAGCTGA-3′  R: 5′-GACACCTTTGTCTTGGAGCTTA-3′ | NM_012854.2 |
| *Nos2* | F: 5′-GAGACGCACAGGCAGAGG-3′  R: 5′-CAGGCACACGCAATGATGG-3′ | NM_001429940.1 |
| **Mouse primers** | | |
| Gene | Primer sequence | Accession No. |
| *Gapdh* | F: 5′-AGGTCGGTGTGAACGGATTTG -3′  R: 5′-TGTAGACCATGTAGTTGAGGTCA -3′ | NM_008084.4 |
| *Rplp0* | F: 5′- CTTCATTGTGGGAGCAGACA -3′  R: 5′- ATGTGAGGCAGCAGTTTCTC -3′ | NM_007475.5 |
| *Bmp2* | F: 5′-GAGGAGGAGGCGAAGAAAAG-3′  R: 5′-AGCAACACTAGAAGACAGCG-3′ | NM_007553.3 |
| *Bsp* | F: 5′-AAACGGTTTCCAGTCCAGGG-3′  R: 5′-AAGTCTCCTCTTCCTCCCCC-3′ | NM_008318.3 |
| *Ocn* | F: 5′- TTCTGCTCACTCTGCTGACC -3′  R: 5′- GGGACTGAGGCTCCAAGGTA -3′ | NM_007541.3 |
| *Nfic* | F: 5′-CACTCCTGTCACTTGTAGGC-3′  R: 5′-ACTCTTTGATGGTCCCAGGA-3′ | NM_008688.3 |
| *Klf4* | F: 5′-CCTTTCAGTGCCAGAAGTGT-3′  R: 5′-CGTGGGAAGACAGTGTGAAA-3′ | NM_010637.3 |
| *Runx2* | F: 5′-TTCTCCAACCCACGAATGCAC-3′  R: 5′-CAGGTACGTGTGGTAGTGAGT-3′ | NM_001146038.1 |
| *Dspp* | F: 5′-AACACATCCAGGAACTGCAGCACA-3′  R: 5′-TGACTCGGAGCCATTCCCATCTCT-3′ | NM_010080.3 |
| *Osx* | F: 5′-CTGCCTGACTCCTTGGGACC-3′  R: 5′-GGGGACTGGAGCCATAGTGA-3′ | NM_130458.4 |
| *Tgfb1* | F: 5′-CTGCTGACCCCCACTGATAC-3′  R: 5′-GGGGCTGATCCCGTTGATT-3′ | NM_011577.2 |
| *Nos2* | F: 5′-GGTGAAGGGACTGAGCTGTT-3′  R: 5′-ACGTTCTCCGTTCTCTTGCAG-3′ | NM_010927.4 |
| *Tnfa* | F: 5′-TAGCCCACGTCGTAGCAAAC-3′  R: 5′-GCAGCCTTGTCCCTTGAAGA-3′ | NM_013693.3 |
| *Il1b* | F: 5′-TGCCACCTTTTGACAGTGATG-3′  R: 5′-TGATGTGCTGCTGCGAGATT-3′ | NM_008361.4 |
| *Arg1* | F: 5′-GTACATTGGCTTGCGAGACG-3′  R: 5′-ATCGGCCTTTTCTTCCTTCCC-3′ | NM_007482.3 |
| *Mrc1* | F: 5′-TTCAGCTATTGGACGCGAGG-3′  R: 5′-GAATCTGACACCCAGCGGAA-3′ | NM_008625.2 |
| *Chil3* | F: 5′-TGGAATTGGTGCCCCTACAA-3′  R: 5′-ACCAACCCACTCATTACCCT-3′ | NM_009892.4 |

**Fig. S1.** Experimental workflow and study design

**
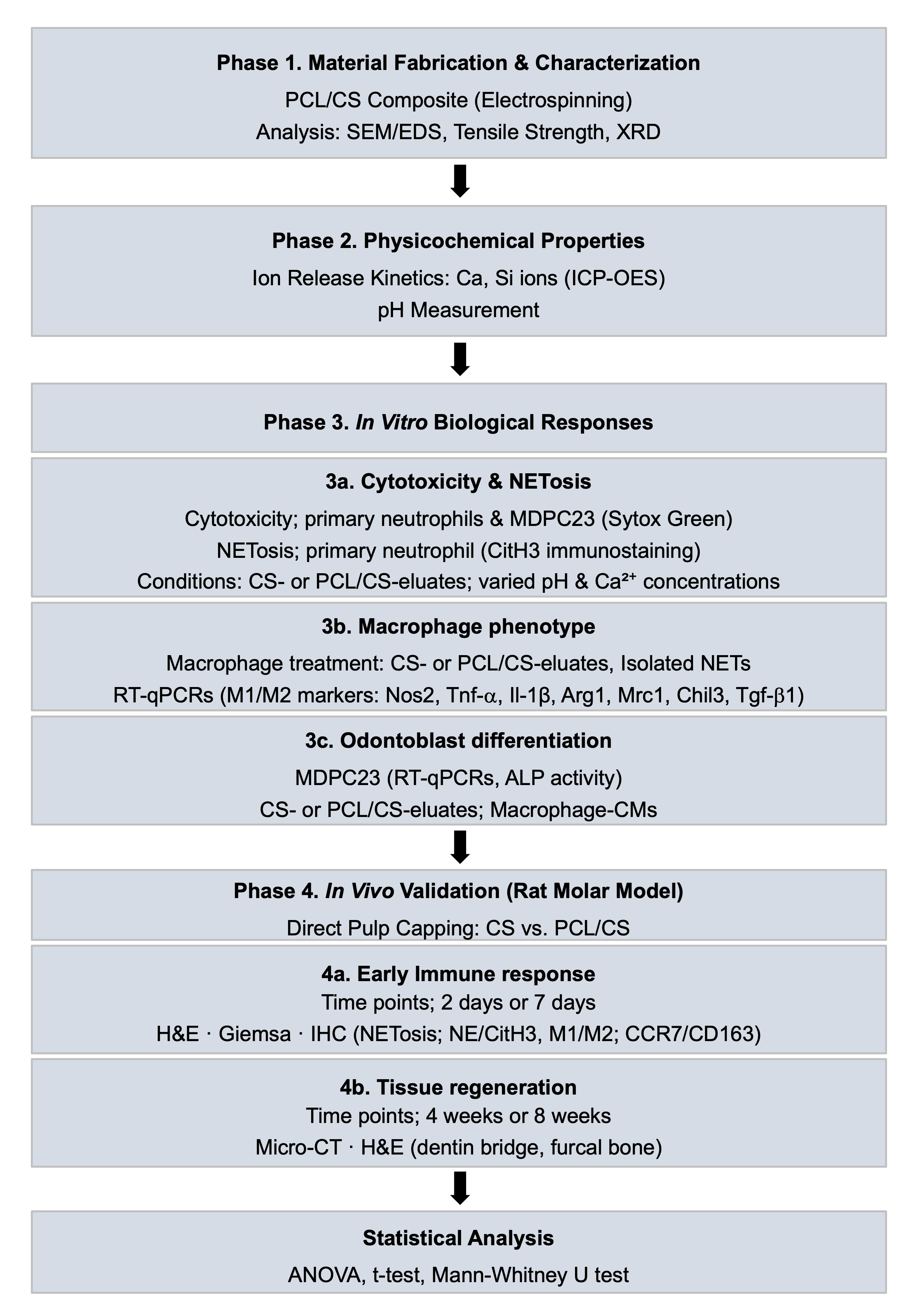
**


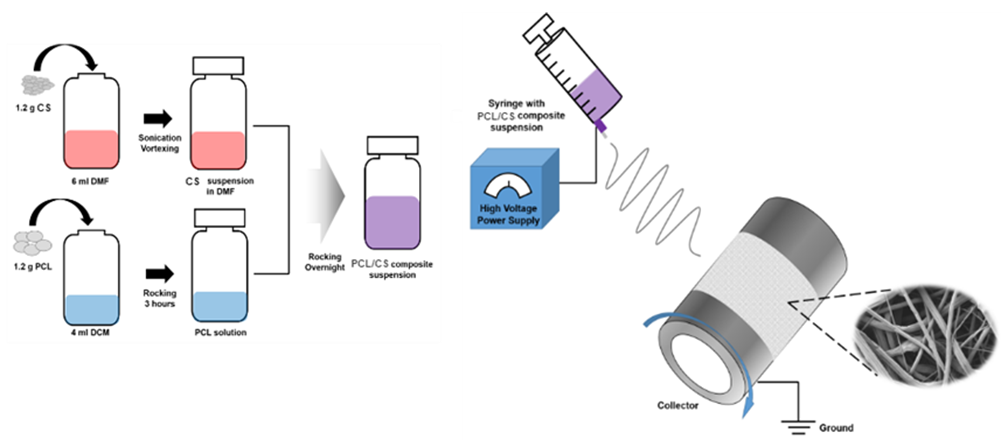


**Fig. S2.** Diagram of the fabrication procedures of PCL/CS.


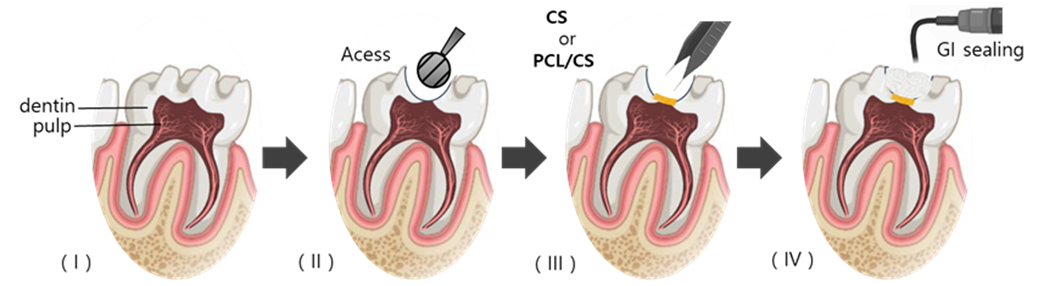


**Fig. S3.** Diagram of an experimental pulp capping performed in the first molars of rats.


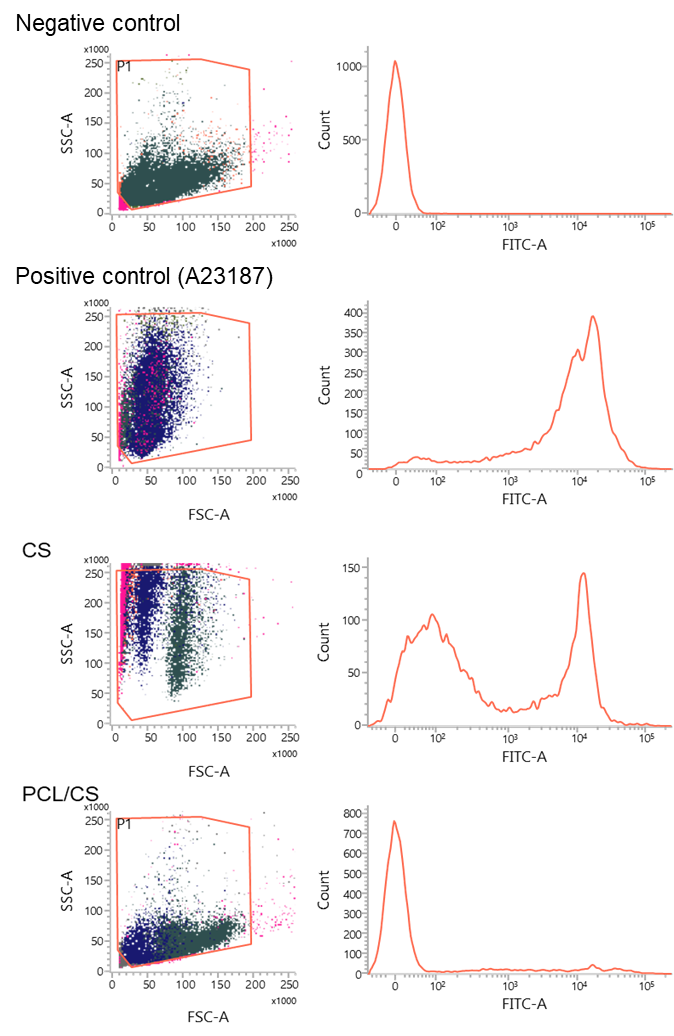


**Fig. S4.** Flow cytometric analysis of SytoxGreen-stained neutrophils treated with D1 CS or PCL/CS eluates (50% v/v).
